# Effects of Different Drying Methods on the Flavor Characteristics and Chemical Profile of *Forsythia suspensa* Flowers Using Electronic Sensors and Mass Spectrometry

**DOI:** 10.3390/foods15101757

**Published:** 2026-05-15

**Authors:** Qingling Xie, Jiangyi Luo, Ling Liang, Wei Su, Mengying Lyu, Caiyun Peng, Bin Li, Wei Wang, Hanwen Yuan

**Affiliations:** TCM and Ethnomedicine Innovation & Development International Laboratory, School of Pharmacy, Hunan University of Chinese Medicine, Changsha 410208, China; xieql12@126.com (Q.X.); jyiluo1998@163.com (J.L.); lliang901@stu.hnucm.edu.cn (L.L.); suwei0310@163.com (W.S.); dancytime@163.com (M.L.); 002142@hnucm.edu.cn (C.P.); libin@hnucm.edu.cn (B.L.)

**Keywords:** Forsythiae Flos, drying treatments, electronic nose, electronic tongue, HS-GC-MS, odor activity value, LC-MS, sensory evaluation

## Abstract

*Forsythia suspensa* flowers are a promising raw material for herbal infusions, but the effects of drying on their flavor and chemical composition remain unclear. Four drying methods, freeze-drying (FD), indoor shade drying (ID), sun drying (SD), and hot-air drying (HAD), were evaluated using an electronic nose, an electronic tongue, HS-GC-MS, LC-MS, sensory evaluation, and correlation analyses. Significant differences in aroma, taste, and overall acceptability scores were observed between drying treatments. HAD samples showed stronger sweetness, bitterness, and umami responses, whereas FD samples showed higher W1W (mainly responsive to terpenes) and W2W (mainly responsive to aromatic compounds) sensor responses. In total, 72 volatile and 148 non-volatile compounds were identified. Aldehydes were the main volatile class, showing the highest relative abundance in SD, whereas terpenes were highest in HAD. OAV analysis revealed 38 volatile compounds with OAV > 1, with nonanal as the major contributor in all groups. LC–MS screened 62 differential non-volatile compounds across the four drying treatments. Pairwise comparisons with FD showed 46 differential compounds, with HAD showing the most distinct changes. Overall, the flavor differences across drying treatments were closely associated with changes in volatile and non-volatile compounds, and HAD showed better potential for standardized processing.

## 1. Introduction

*Forsythia suspensa* is a widely used medicinal plant, and its fruits have been extensively studied for their medicinal value [1,2]. The flowers have also attracted interest for potential use in herbal infusions and dried floral products because of their appealing appearance and pleasant aroma after drying [3,4,5]. In addition, the flowers contain various bioactive constituents, including phenolics such as flavonoids, phenylethanoid glycosides, and amino acids, which may contribute to their sensory properties [4,6,7]. However, compared with the fruits of *Forsythia suspensa,* the flowers have received much less attention regarding flavor characteristics and chemical composition under different drying methods [4,6]. Aroma and taste are especially important in infusions, herbal preparations, and dried floral ingredients because they strongly influence sensory quality and consumer acceptance [5]. Therefore, further investigation is needed to clarify how different drying methods influence the flavor characteristics of *Forsythia suspensa* flowers and their potential utilization in food and infusion products.

Drying is an essential postharvest treatment for botanical materials because it reduces moisture content, improves storage stability, and facilitates subsequent storage, and transportation. It also significantly affects flavor quality by altering both volatile and non-volatile compounds [8,9]. Different drying methods involve different temperatures, dehydration rates, and oxygen exposures, which can affect the retention, degradation, and formation of compounds associated with aroma and taste [10,11]. Freeze drying is often considered suitable for preserving temperature sensitive compounds, whereas hot-air drying offers advantages in process control and large-scale application. Sun drying and indoor shade drying are simpler and less costly alternatives, but their drying conditions are generally less controllable [12,13]. Consequently, variations in aldehydes, alcohols, esters, terpenes, organic acids, phenolics, and other compounds may lead to differences in flavor characteristics [14,15]. Therefore, comparing drying methods is important for evaluating sensory changes and clarifying the relationships between flavor characteristics and chemical composition.

The flavor characteristics of dried botanical materials are shaped by both volatile and non-volatile compounds [16,17]. Volatile compounds are associated with aroma, whereas non-volatile compounds, particularly phenolics and amino acids, are more closely associated with bitterness, sweetness, and astringency [18,19,20]. In recent years, intelligent sensory technologies such as the electronic nose (E-nose) and electronic tongue (E-tongue), together with gas chromatography–mass spectrometry and liquid chromatography–mass spectrometry, have been widely used for flavor characterization of botanical materials and food products [21,22]. These approaches enable comprehensive sensory evaluation, identification of key volatile and non-volatile compounds, and discrimination of differences among treatments through multivariate statistical analysis [23,24]. Nevertheless, studies on flavor changes caused by different drying methods in botanical materials often focus only on volatile compounds or a single aspect of flavor [9,25]. Comprehensive investigations linking aroma, taste, volatile profiles, non-volatile profiles, and their relationships are still limited. In particular, systematic evidence is still lacking on how drying methods reshape the flavor characteristics of *Forsythia suspensa* flowers through combined changes in aroma-active compounds and compounds associated with taste attributes. This limitation hinders the selection of suitable drying methods for improving flavor characteristics and the further utilization of *Forsythia suspensa* flowers in food and infusion products.

Therefore, this study aimed to systematically evaluate the effects of four drying methods, namely freeze drying (FD), indoor shade drying (ID), sun drying (SD), and hot-air drying (HAD), on the flavor characteristics of *Forsythia suspensa* flowers. An integrated analytical strategy combining E-nose, E-tongue, HS-GC-MS, LC-MS, odor activity value analysis, sensory evaluation, multivariate statistics, and correlation analysis was employed to characterize aroma, taste, volatile profiles, non-volatile profiles, and their relationships among the drying treatments. Specifically, this study addresses the following gaps:

- The effects of drying methods on the flavor characteristics of *Forsythia suspensa* flowers for herbal infusions remain insufficiently understood.
- Changes in volatile and non-volatile profiles under different drying methods have not been sufficiently interpreted in relation to aroma and taste responses.
- The resulting combined evidence can support the selection of suitable drying methods and improve standardized processing of *Forsythia suspensa* flowers.

## 2. Materials and Methods

### 2.1. Reagents and Chemicals

Methyl nonanoate (≥99.8%, GC) was used as the internal standard and purchased from Sigma-Aldrich (St. Louis, MO, USA). The *n*-alkane calibration standard (C7–C40) and *n*-hexane (GC grade, ≥99.0%) were purchased from Merck (Darmstadt, Germany), and ultrapure water was obtained from a Milli-Q Synthesis system (Millipore, Bedford, MA, USA).

### 2.2. Sample Collection and Drying Processes

Fresh *Forsythia suspensa* flowers (5 kg) were collected at the full-bloom stage in early April from the same cultivation area in Linfen, Shanxi Province, China, to minimize variation caused by origin and developmental stage. After removal of impurities, damaged materials, and immature flowers, the remaining flowers were pooled and gently mixed to ensure uniformity. This study was therefore conducted on a single biological batch.

A single-factor experimental design was used, with drying treatment as the experimental factor and four treatment levels: indoor shade drying (ID), sun drying (SD), hot-air drying (HAD), and freeze-drying (FD). The pooled flower samples were divided into four portions and subjected to the different drying treatments. Indoor shade drying (ID) was performed at 20–25 °C for 3 days in an indoor environment with natural air circulation and no direct sunlight. Sun drying (SD) was performed under natural sunlight for approximately 10 h until the samples were sufficiently dried for subsequent grinding. According to the nearest available local meteorological records, the estimated relative humidity during SD was approximately 19–56%. Light intensity and UV radiation were not directly measured at the drying site, and SD was therefore defined as natural sunlight exposure. Hot-air drying (HAD) was conducted at 60 °C for approximately 5 h until the samples reached a stable weight. The temperature of 60 °C was selected with reference to previous studies on flowers and flower teas in which hot-air or oven drying was applied [8,12]. Freeze-drying (FD) was performed at −50 °C for 24 h. Freeze-drying was conducted using a SCIENTZ-10N vacuum freeze dryer (Ningbo Scientz Biotechnology Co., Ltd., Ningbo, China), and hot-air drying was carried out in a 101-2AB electric blast drying oven (Tianjin Taisite Instrument Co., Ltd., Tianjin, China). After drying, the samples were ground into powder, passed through a 60-mesh sieve, sealed, and stored at −20 °C for subsequent analyses. For each drying treatment, three analytical replicates were prepared from the corresponding dried powder and used for subsequent analyses.

### 2.3. Electronic Nose Analysis

An electronic nose analysis was performed using a PEN3 electronic nose system (Airsense Analytics GmbH, Schwerin, Germany), which was equipped with a ten-sensor metal oxide semiconductor (MOS) array with different selectivities. The powdered sample (0.5 g) was accurately weighed into a 20 mL headspace vial and immediately sealed with a PTFE-silicone septum. The sealed vials were incubated in a water bath at 60 °C for 30 min to allow volatile compounds to equilibrate in the headspace. Subsequently, the headspace gas was introduced into the sensor chamber using clean air as the carrier gas, with both the injection flow rate and chamber flow rate set at 400 mL/min.

The acquisition parameters were set as follows: sampling interval, 1 s; pre-sampling time, 5 s; and measurement time, 60 s. After each measurement, the sensor chamber was purged with clean air for 60 s to restore the sensor baseline before the next analysis. No dilution of the headspace gas was applied during the analysis. All measurements were performed in triplicate under identical conditions. For data processing, the stable response region of each sensor signal was selected, and the mean response value of each sensor within this region was extracted for subsequent multivariate statistical analysis and comparison among different drying treatments.

### 2.4. Electronic Tongue Analysis

The taste characteristics of *Forsythia suspensa* flower samples were analyzed using a TS-5000Z electronic tongue system (Insent Inc., Atsugi, Japan), equipped with sensors for umami (AAE), sourness (CA0), saltiness (CT0), bitterness (C00), astringency (AE1), and sweetness (GL1), as well as outputs for astringent aftertaste (Aftertaste-A, AE1), bitter aftertaste (Aftertaste-B, C00), and richness (umami aftertaste, AAE).

For sample preparation, 1.5 g of dried flower powder was infused with 150 mL of boiled deionized water at a solid-to-liquid ratio of 1:100 (*w*/*v*), and the infusion was then filtered to obtain a tea-like sample solution. After cooling to 25 ± 2 °C, 35 mL of the filtrate was transferred into the sample cup for electronic tongue analysis. Each measurement consisted of three sequential steps: taste detection for 30 s, aftertaste detection for 30 s, and sensor cleaning for 120 s. For each sample, the stable sensor response values recorded during the taste detection stage were extracted for subsequent statistical analysis. All measurements were performed in triplicate under identical conditions.

### 2.5. HS-GC-MS Analysis of Volatile Compounds

#### 2.5.1. Sample Preparation and HS-GC-MS Analysis

Approximately 0.2 g of powdered *Forsythia suspensa* flower sample was accurately weighed into a 20 mL headspace vial. Subsequently, 10 μL of methyl nonanoate internal standard solution (0.1 mg/mL in n-hexane) was added, and the vial was immediately sealed. The sample was equilibrated at 120 °C for 15 min using an Agilent 7697A headspace sampler (Agilent Technologies, Santa Clara, CA, USA). The temperatures of the sample loop and transfer line were set at 130 °C and 140 °C, respectively, and the loop hold time was 0.05 min. Thereafter, 1000 μL of headspace gas was injected into the GC inlet at a split ratio of 1:5.

GC-MS analysis was carried out using an Agilent 7890B gas chromatograph coupled with an Agilent 7000A mass spectrometer (Agilent Technologies, Santa Clara, CA, USA), equipped with an HP-5MS capillary column (30 m × 0.25 mm i.d., 0.25 μm film thickness). The oven temperature program was as follows: initially held at 50 °C for 3 min, ramped to 80 °C at 3 °C/min and held for 2 min, then increased to 120 °C at 5 °C/min and held for 2 min, and finally raised to 240 °C at 12 °C/min. A post-run hold was performed at 280 °C for 3 min. The injector temperature was maintained at 250 °C. The mass spectrometer was operated in electron ionization (EI) mode at 70 eV. The ion source and quadrupole temperatures were set at 250 °C and 150 °C, respectively. Full-scan mass spectra were acquired over an m/z range of 50–550, with a solvent delay of 4 min.

#### 2.5.2. Qualitative and Quantitative Analysis of Volatile Compounds

Volatile compounds were identified by comparing their mass spectra with the NIST 17 mass spectral library, combined with retention index (RI) matching. Retention indices were calculated under identical chromatographic conditions using a homologous series of n-alkanes (C7–C40) and compared with reference RI values reported in databases and the literature, with an acceptance tolerance of ±20.

Methyl nonanoate was selected as the internal standard because it was absent from the sample matrix, possessed appropriate volatility, and exhibited good chromatographic resolution without co-elution with endogenous volatile compounds. Volatile compounds were semi-quantified using the internal standard method, and the content of each compound was calculated according to the following equation:

(1) $$C_{i}\left(\mu g/g\right)=\frac{A_{i}}{A_{IS}}\times \frac{C_{IS}}{m}\times V_{IS}$$

where $A_{i}$ and $A_{IS\;}$ are the peak areas of the analyte and the internal standard, respectively; $C_{IS}$ is the concentration of the internal standard solution (µg/mL); $V_{IS}$ is the volume of internal standard added (mL); and m is the mass of the sample (g). The results were expressed as μg/g. All analyses were performed in triplicate.

#### 2.5.3. Odor Activity Value (OAV) Calculation

Odor activity values (OAVs) were calculated to evaluate the contribution of individual volatile compounds to the overall aroma profile of *Forsythia suspensa* flower tea. The OAV of each compound was defined as the ratio of its relative concentration to its corresponding odor threshold, according to the following equation:

(2) $${OAV}_{i}=\frac{C_{i}}{{OT}_{i}}$$

where $C_{i}$ represents the relative concentration of compound i, determined by HS-GC–MS using the internal standard method, and ${OT}_{i}$ denotes the corresponding odor threshold value in water. Odor threshold values were obtained from the published literature and established odor databases under comparable conditions. Because these odor threshold values were mainly obtained in water, the calculated OAVs were used as approximate indicators of aroma contribution. Compounds with OAV > 1 were considered aroma-active compounds and were selected for further analysis and discussion.

### 2.6. LC-MS Analysis of Non-Volatile Compounds

#### 2.6.1. Sample Preparation and LC-MS Analysis

LC-MS data acquisition was conducted according to a previously reported method with minor modifications [26]. Briefly, powdered samples (50 mg) were extracted with 50 mL of 70% methanol by ultrasonication for 30 min and then centrifuged at 13,000 rpm for 10 min. The supernatants were collected for LC-MS analysis. Quality control (QC) samples were prepared by pooling equal volumes of all extracts and were used to monitor the stability of the analytical system. All extracts were stored at −40 °C prior to analysis. Chromatographic separation was performed on a Hypersil GOLD™ Aq-C18 column (100 × 2.1 mm, 1.9 μm; Thermo Fisher Scientific, Waltham, MA, USA). The mobile phases consisted of 0.1% formic acid in water (A) and acetonitrile (B). The flow rate was 0.3 mL/min, the column temperature was maintained at 30 °C, and the injection volume was 2 μL. The gradient elution program is shown in Table S1.

MS detection was performed on an Orbitrap Exploris 120 mass spectrometer (Thermo Fisher Scientific, Waltham, MA, USA) equipped with an H-ESI source and operated in both positive and negative ion modes. The spray voltages were set at 3.5 kV and −3.0 kV for the positive and negative modes, respectively. The ion transfer tube temperature was 325 °C, and the vaporizer temperature was 350 °C. Data were acquired over an m/z range of 100–1200. MS/MS spectra were obtained in ddMS2 mode using stepped HCD collision energies of 30, 60, and 90 eV. QC samples were injected at the beginning of the run and after every eight sample injections to evaluate instrumental stability.

#### 2.6.2. Data Analysis and Processing

Raw LC-MS data were processed using Compound Discoverer 3.3 (Thermo Fisher Scientific, Waltham, MA, USA), including peak extraction, baseline correction, retention time alignment, background subtraction, gap filling, and peak area normalization. Positive- and negative-ion data were processed separately. Features were extracted within a retention time range of 1–60 min using a mass tolerance of 5 ppm and a minimum intensity threshold of 100,000. Only features with MS/MS spectra were retained for subsequent compound annotation.

Compound annotation was performed based on a previously reported strategy with slight modifications [26]. Compounds were tentatively annotated by integrating accurate mass measurements, retention behavior, and MS/MS fragmentation patterns through comparison with mzCloud, ChemSpider, other publicly available databases, self-constructed databases, and published literature. Annotation confidence was assigned according to the Metabolomics Standards Initiative (MSI) [27]. Compounds were assigned as MSI Level 1 only when their retention time, accurate mass, and MS/MS spectra matched those of authentic standards in an in-house library established under the same LC-MS/MS conditions. Compounds putatively annotated based on accurate mass, MS/MS fragmentation patterns, database matching, and literature information were assigned as MSI Level 2. Compounds assigned only to a compound class or structural type based on accurate mass and partial fragment information were assigned as MSI Level 3. Features without reliable structural information or compound class information were considered MSI Level 4 and were not included in the list of annotated compounds. Relative abundances were expressed as normalized peak areas.

### 2.7. Sensory Evaluation

A preliminary sensory evaluation was performed by six participants (three males and three females, aged 21–24 years) to provide supplementary sensory information for interpreting the instrumental results. A brief familiarization session was conducted before the evaluation, during which the evaluation procedure and scoring criteria were explained to the participants. To minimize the influence of environmental factors on the evaluation results, the sensory test was performed in a quiet, odor-free room under controlled conditions. Participants were instructed to refrain from eating for at least 1 h before the session, and communication among them was not permitted during evaluation.

Briefly, 3.0 g of dried *Forsythia suspensa* flowers were infused with 100 mL of freshly boiled water for 5 min, and the infusion was filtered immediately for sensory evaluation. Each infusion was allowed to cool under the same conditions and then presented in identical containers labeled with randomly assigned three-digit codes. The presentation order was randomized for each participant. Water was provided as a palate cleanser between samples.

Sensory properties were evaluated in terms of appearance, infusion color, aroma, taste, and overall acceptability. Infusion color, aroma, and taste were rated using a 0–9 intensity scoring scale (0 = not perceptible; 9 = extremely strong). Appearance and overall acceptability were evaluated using a 9-point acceptability scale. Each sample was assessed independently by all participants. All participants provided informed consent before the sensory evaluation.

### 2.8. Statistical Analysis

All experiments were performed in triplicate, and the results are expressed as mean ± standard deviation (SD). Statistical analysis was conducted using SPSS 26.0 (IBM, Armonk, NY, USA). The effect of drying treatment on each measured variable was assessed using one-way analysis of variance (ANOVA), and group means were compared using Duncan’s multiple range test. Statistical significance was set at *p* < 0.05.

Principal component analysis (PCA), partial least squares discriminant analysis (PLS-DA), variable importance in projection (VIP) analysis, and permutation tests were performed using SIMCA 14.1 (Umetrics, Umeå, Sweden). For differential compound screening, *p*-values from one-way ANOVA across the four drying treatments were adjusted using the Benjamini–Hochberg false discovery rate (FDR) method and reported as q-values. For the overall comparison of volatile and non-volatile compounds, variables with VIP > 1 and q-value < 0.05 were considered differential compounds. For pairwise comparisons of non-volatile compounds, pairwise *p*-values were also adjusted using the Benjamini–Hochberg FDR method, and variables with VIP > 1.3, q-value < 0.05, and |log_2_FC| ≥ 1 were considered differential compounds.

Venn diagrams, boxplots, and volcano plots were generated using the MetWare platform. Spearman correlation analysis and correlation heatmaps were performed using R software (version 4.3.1). Total ion chromatograms (TICs) and the remaining figures were generated using Origin 2024b (OriginLab, Northampton, MA, USA).

## 3. Results and Discussion

### 3.1. Electronic Nose Analysis

**Figure 1:**
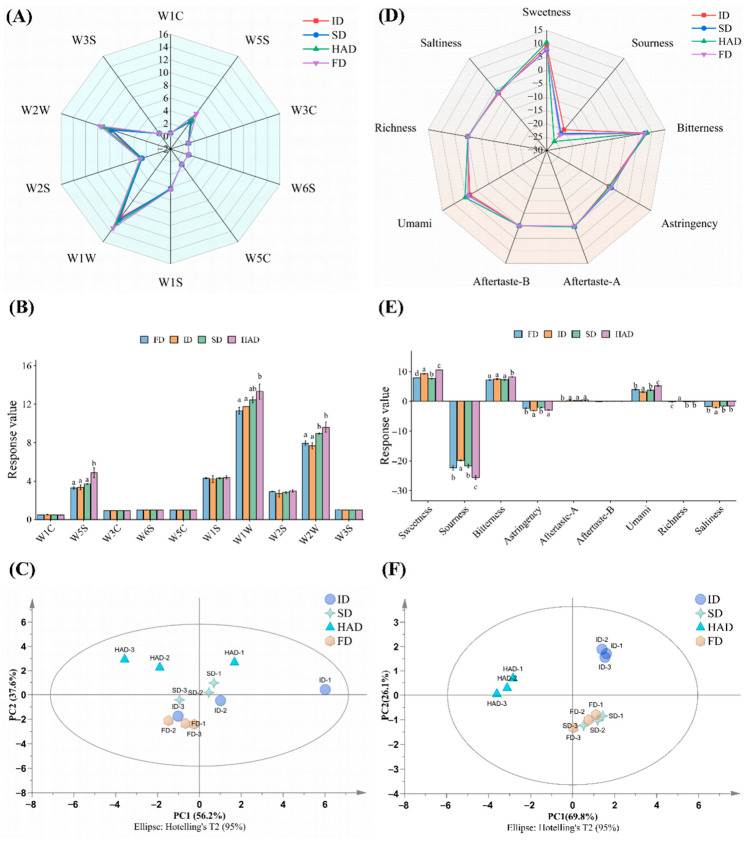
Electronic nose and electronic tongue analyses of *Forsythia suspensa* flowers subjected to different drying methods. (**A**) Radar plot of electronic nose sensor responses; (**B**) Response value analysis of electronic nose sensors; (**C**) PCA score plot based on electronic nose; (**D**) Radar plot of electronic tongue taste attributes; (**E**) Response value analysis of electronic tongue taste attributes; (**F**) PCA score plot based on electronic tongue. ID, indoor shade drying. SD, sun drying. HAD, hot-air drying. FD, freeze-drying. Aftertaste-A indicates astringent aftertaste, and Aftertaste-B indicates bitter aftertaste. Different lowercase letters indicate significant differences between treatments according to Duncan’s multiple range test (*p* < 0.05).

The aroma characteristics of *Forsythia suspensa* flowers processed by different drying methods were evaluated using a PEN3 electronic nose, and the results are presented in Figure 1A–C. According to the E-nose response patterns shown in Figure 1A,B, the four drying treatments produced distinct volatile profiles. Among all sensors, W1W (sensitive to sulfur-containing organic compounds), W2W (sensitive to aromatic compounds and organic sulfides), and W5S (sensitive to nitrogen oxides) showed the strongest responses. The FD group exhibited the highest responses at W1W and W2W, whereas the HAD group showed the highest response at W5S, suggesting that sulfur-containing volatiles, aromatic compounds, and nitrogen oxide-related compounds may contribute importantly to the differences among treatments. In contrast, the responses of W1C, W3C, W6S, W5C, and W3S were comparatively weak and varied only slightly between the treatment groups. Therefore, the E-nose results indicated clear differences in volatile response patterns between the drying treatments, which were further characterized by HS-GC-MS.

The PCA score plot (Figure 1C) showed that the first two principal components accounted for 93.8% of the total variance, with PC1 and PC2 explaining 56.2% and 37.6%, respectively. The four drying groups were well separated, and the replicate samples within each group were closely clustered. ID was mainly located on the positive side of PC1, HAD on the negative side of PC1, FD on the negative side of PC2, and SD in the central region. The PLS-DA score plot (Figure S1A) further increased the separation among the groups. The VIP plot (Figure S1B) showed that W2W, W1W, W1S, and W5S had VIP values greater than 1, with W2W showing the highest VIP value.

### 3.2. Electronic Tongue Analysis

Electronic tongue analysis was performed to compare the taste profiles of *Forsythia suspensa* flowers subjected to different drying methods (Figure 1D–F). As shown in Figure 1D,E, the response values for sweetness, sourness, bitterness, umami, astringency, saltiness, and aftertaste-related attributes differed among the drying treatments. All samples showed pronounced bitterness and negative sourness responses. The HAD samples exhibited higher response values for sweetness, bitterness, and umami, together with the lowest sourness response. The FD samples showed relatively low response values for sweetness and umami. The ID and SD samples were distributed at intermediate levels. In contrast, Aftertaste-A, Aftertaste-B, astringency, and saltiness changed only slightly among the treatments.

The PCA score plot (Figure 1F) showed that the first two principal components accounted for 95.9% of the total variance, with 69.8% explained by PC1 and 26.1% by PC2. The four treatment groups were clearly separated, and good clustering of replicates was observed within each group. ID was mainly distributed in the positive regions of both PC1 and PC2, HAD was located on the negative side of PC1, and FD and SD were positioned close to each other. The PLS-DA score plot (Figure S1C) showed a clearer separation of HAD from the remaining treatments, while ID also remained separated from FD and SD. The VIP plot (Figure S1D) showed that sourness, sweetness, aftertaste, and astringency all had VIP values greater than 1, with sourness ranking first, followed by sweetness, aftertaste, and astringency.

### 3.3. HS-GC-MS Analysis of Volatile Compounds

#### 3.3.1. Identification of Volatile Compounds

**Figure 2:**
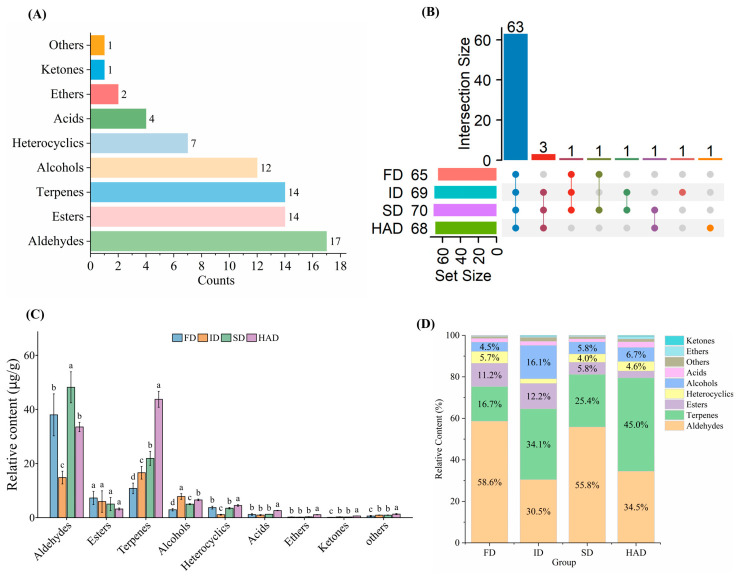
HS-GC–MS analysis of volatile compounds in *Forsythia suspensa* flowers subjected to different drying methods. (**A**) Distribution of volatile compounds among different chemical classes; (**B**) UpSet plot of shared and unique volatile compounds among different drying treatments. (**C**) Relative contents of different classes of volatile compounds; (**D**) Relative proportions of different classes of volatile compounds. FD, freeze-drying; ID, indoor shade drying; SD, sun drying; HAD, hot-air drying. Different lowercase letters indicate significant differences between treatments according to Duncan’s multiple range test (*p* < 0.05).

The HS-GC-MS total ion chromatograms of *Forsythia suspensa* flowers subjected to different drying methods (Figure S2A) showed obvious differences in peak intensity and distribution, indicating that the drying method affected the composition of volatile compounds. As shown in Table S2, a total of 72 volatile compounds were identified, including aldehydes (17), esters (14), terpenes (14), alcohols (12), heterocyclic compounds (7), acids (4), ethers (2), ketones (1), and others (1). The distribution of volatile compound classes (Figure 2A) showed that aldehydes were the most abundant class, followed by esters and terpenes.

The UpSet plot of volatile compounds (Figure 2B) revealed substantial overlap among the four groups, with 63 volatile compounds commonly detected in all treatments, indicating that shared compounds dominated the volatile profile. In contrast, only a few compounds were specific to individual treatments. Tridecanal, tetradecanal, pentadecanal, and hexadecanal were detected only in FD. α-Thujene and 1-pentanol were detected only in ID. 2,4-Dihydroxy-6-methylbenzaldehyde was detected only in SD. Trans-2,4-dimethyloxetane was detected only in HAD. The numbers of volatile compounds identified under different drying methods were 65 (FD), 69 (ID), 70 (SD), and 68 (HAD), indicating that the drying method affected both the number and composition of volatile compounds.

The relative contents and proportions of different classes of volatile compounds varied markedly among the drying treatments (Figure 2C,D). Aldehydes remained dominant in all four groups, with the highest total content in SD (48.18 μg/g), followed by FD (38.00 μg/g) and HAD (33.52 μg/g), whereas ID showed a much lower level (14.82 μg/g). Consistent with this pattern, aldehydes accounted for 58.6% and 55.8% of the total volatile compounds in FD and SD, respectively. Terpenes were most abundant in HAD, reaching 43.75 μg/g and accounting for 45.0% of the total volatile compounds. Alcohols showed the highest total content in ID (7.82 μg/g), with a proportion of 16.1%. Esters varied only slightly among the four treatments, whereas heterocyclic compounds were also higher in HAD than in ID. Overall, SD was characterized by aldehyde predominance, HAD by terpene enrichment, and ID by a relatively higher proportion of alcohols.

The distribution of representative individual compounds was consistent with the class differences. Hexanal and heptanal showed relatively higher levels in SD, whereas nonanal reached 16.21 μg/g in SD and was one of the most abundant aldehydes. Among terpenes, α-pinene, sabinene, and β-pinene were most abundant in HAD, reaching 7.16, 7.95, and 21.94 μg/g, respectively. Among alcohols, phenylethyl alcohol showed relatively higher levels in ID and HAD. Among heterocyclic compounds, pyranone was markedly higher in HAD (1.88 μg/g) than in ID (0.060 μg/g). Overall, these results further confirmed aldehyde predominance in SD, terpene enrichment in HAD, and a relatively higher contribution of alcohols in ID.

#### 3.3.2. Multivariate Analysis of Volatile Compounds

**Figure 3:**
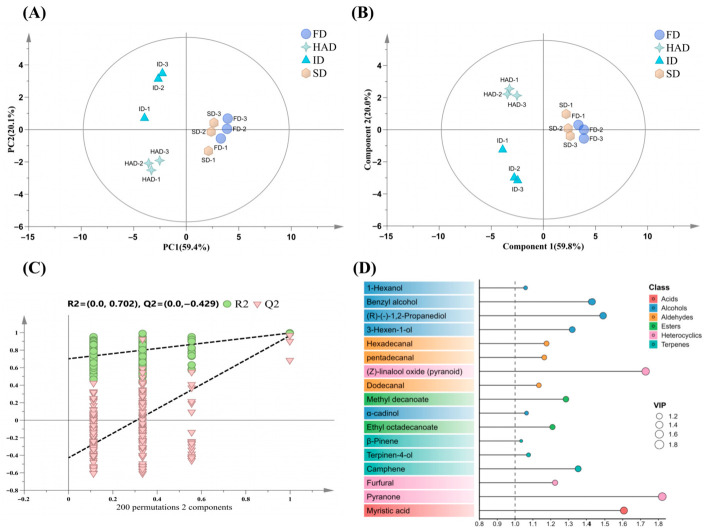
Multivariate analysis of volatile compounds in *Forsythia suspensa* flowers under different drying methods. (**A**) PCA score plot; (**B**) PLS-DA score plot; (**C**) Permutation test of the PLS-DA model; (**D**) VIP values of the 17 differential volatile compounds screened based on VIP > 1 and q-value < 0.05. The dashed line indicates VIP = 1. FD, freeze-drying; ID, indoor shade drying; SD, sun drying; HAD, hot-air drying.

To further evaluate the overall variation in volatile compounds among *Forsythia suspensa* flowers processed under different drying methods, multivariate analysis was carried out using the HS-GC-MS-derived volatile compound data. PCA was first used as an unsupervised method to provide an overview of sample distribution. The PCA score plot (Figure 3A) showed that the first two principal components together explained 79.5% of the total variance, with 59.4% attributed to PC1 and 20.1% to PC2. The samples tended to group according to drying treatment, and replicates from the same treatment were generally close to each other, suggesting that drying affected the overall volatile profile. PLS-DA was then used as a supervised exploratory analysis to further examine group separation (Figure 3B). The model showed R^2^X = 0.952, R^2^Y = 0.989, and Q^2^ = 0.919, suggesting acceptable model fit and predictive performance. In the permutation test (Figure 3C), the R^2^ and Q^2^ intercepts were 0.702 and −0.429, showing that the model did not exhibit obvious overfitting. The VIP values derived from PLS-DA were subsequently combined with q-values obtained from FDR-adjusted one-way ANOVA to screen differential volatile compounds that differed across drying treatments.

The VIP plot (Figure 3D) presented the 17 differential volatile compounds screened based on VIP > 1 and q-value < 0.05 across the four drying treatments, and detailed information is provided in Table S4. Among these compounds, pyranone, (Z)-linalool oxide (pyranoid), myristic acid, (R)-(-)-1,2-propanediol, benzyl alcohol, and camphene showed the highest VIP values. The clustering heatmap of these compounds showed different abundance patterns among the four drying treatments and further indicated changes in individual volatile compounds (Figure S2B). Specifically, HAD showed relatively higher abundances of β-pinene, terpinen-4-ol, camphene, α-cadinol, and ethyl octadecanoate, whereas ID showed relatively higher abundances of 3-hexen-1-ol, benzyl alcohol, and (R)-(-)-1,2-propanediol. FD showed relatively higher abundances of methyl decanoate, furfural, and myristic acid. FD and SD showed relatively higher abundances of several aldehydes, including hexadecanal and pentadecanal, whereas dodecanal was more abundant in SD. Overall, the volatile differences induced by drying involved multiple classes of compounds, including aldehydes, alcohols, terpenes, heterocyclics, and acids.

#### 3.3.3. OAV Analysis of Key Aroma-Active Compounds

To further evaluate the effects of different drying treatments on aroma formation in *Forsythia suspensa* flowers, odor activity values (OAVs) were calculated based on the concentrations of volatile compounds and their odor thresholds (OTs) [28]. As shown in Table 1, 38 compounds exhibited OAVs above 1, including 13 aldehydes, 6 esters, 9 terpenes, 6 alcohols, 2 heterocyclic compounds, and 1 ether. Compounds with OAVs above 1 are generally considered to contribute directly to the overall aroma of a sample and were therefore identified as the key aroma-active compounds in *Forsythia suspensa* flowers [22,29]. However, because most OT values used in this study were determined in water, sensory thresholds may differ in the actual *Forsythia suspensa* flower sample matrix. Therefore, the OAV results should be interpreted as comparative estimates rather than absolute measures of aroma impact.

**Figure 4:**
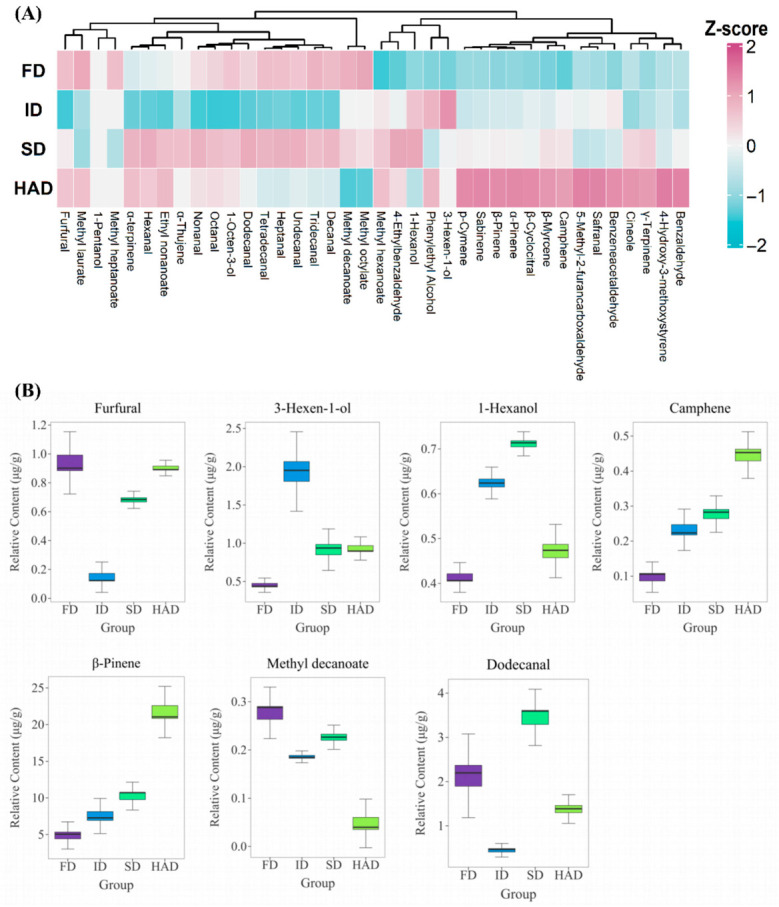
Heatmap and boxplots of key aroma-active compounds in *Forsythia suspensa* flowers under different drying methods. (**A**) Heatmap of key aroma-active compounds with OAV > 1; (**B**) Boxplots of the relative contents of key differential aroma-active compounds. The compounds were selected based on VIP > 1, q-value < 0.05, and OAV > 1. FD, freeze-drying; ID, indoor shade drying; SD, sun drying; HAD, hot-air drying.

The heatmap of aroma-active compounds with OAVs greater than 1 (Figure 4A) showed distinct variation in the major aroma contributors across the four drying treatments. Among these compounds, nonanal showed the highest contribution, with OAVs of 11,080.70, 4818.32, 14,736.05, and 10,910.83 in the FD, ID, SD, and HAD groups, respectively, and the highest value in the SD group [33]. Several other aldehydes, including hexanal, heptanal, octanal, and decanal, also showed relatively high OAVs in SD. Notably, octanal, heptanal, and decanal reached OAVs of 1027.42, 976.49, and 756.87, respectively, further indicating that aldehydes were the dominant aroma-active compounds in the SD group [34]. Based on the odor descriptions of these aldehydes, the SD group was associated with stronger green, fatty, citrus-like, and lemon-like notes, which contributed to its relatively fresh and green aroma profile. These high OAV aldehydes were mainly associated with green, fatty, citrus-like, and lemon-like notes, indicating that the SD group exhibited a more pronounced green and fresh aroma profile [35].

In contrast, the HAD group was mainly characterized by elevated OAVs of several terpenes, including α-pinene, camphene, sabinene, β-pinene, β-myrcene, p-cymene, and γ-terpinene. Among these compounds, β-pinene and β-myrcene reached OAVs of 156.72 and 126.00, respectively, whereas cineole and safranal also showed relatively high OAVs of 302.76 and 268.71. These compounds together indicated a more pronounced pine-like, woody, minty, and camphor-like aroma in the HAD group [36]. By comparison, the ID group showed higher OAVs of alcohols such as 3-hexen-1-ol and phenylethyl alcohol, with values of 27.61 and 5.91, respectively, while 1-hexanol also remained at a relatively high level, suggesting better retention of herbal, green, and partly sweet or floral notes [12]. Although the overall aldehyde contribution in the FD group was lower than that in the SD group, tridecanal and tetradecanal still exhibited OAVs of 11.49 and 36.64, respectively. Methyl heptanoate and methyl decanoate reached OAVs of 322.68 and 31.03, respectively, and methyl laurate also remained at a relatively high level, suggesting a better retention of certain long chain aldehydes and esters in the FD group [37].

The boxplots of the relative contents of key differential aroma-active compounds (Figure 4B) further showed clear differences in representative compounds among the drying methods, mainly including furfural, 3-hexen-1-ol, 1-hexanol, camphene, β-pinene, methyl decanoate, and dodecanal. The changes in the relative contents of these compounds were generally consistent with the contribution trends of the key aroma-active compounds shown in Figure 4A. Specifically, furfural showed high relative levels in the FD and HAD groups. 3-Hexen-1-ol and 1-hexanol were mainly enriched in the ID group. Camphene and β-pinene showed the highest relative abundance in the HAD group. Methyl decanoate had the highest relative content in the FD group. Dodecanal reached its highest relative level in the SD group.

These results indicate that although different drying methods did not change the fact that aldehydes, terpenes, esters, and alcohols remained the main volatile classes in *Forsythia suspensa* flowers, they did affect the relative abundance and aroma contribution of key volatiles. Combined with the multivariate analysis and OAV results, these findings suggest that the aroma differences between the drying methods arose from the combined changes in multiple classes of compounds rather than from a single class, with the HAD group showing the most distinct separation from the other groups. Based on these differences in volatile compounds, LC-MS was further used to characterize the non-volatile compounds in samples subjected to different drying methods.

### 3.4. LC-MS Analysis of Non-Volatile Compounds

#### 3.4.1. Overview of Non-Volatile Compounds

**Figure 5:**
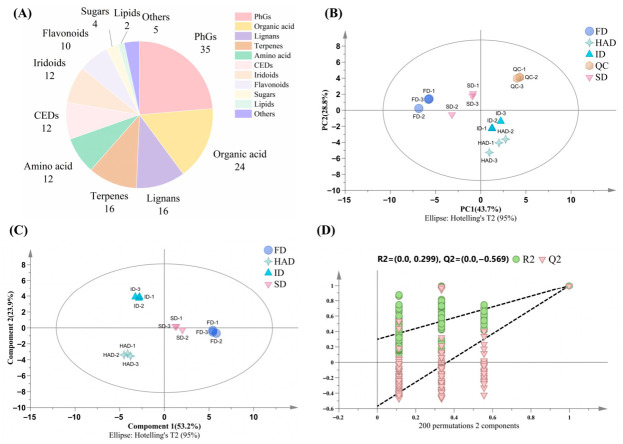
Multivariate statistical analysis of non-volatile compounds in *Forsythia suspensa* flowers under different drying methods. (**A**) Chemical class distribution of identified compounds; (**B**) PCA score plot; (**C**) PLS-DA score plot; (**D**) permutation test of the PLS-DA model. FD, freeze-drying; ID, indoor shade drying; SD, sun drying; HAD, hot-air drying; QC, quality control.

The LC-MS total ion chromatograms (TICs) of *Forsythia suspensa* flowers under different drying treatments are shown in Figure S3. In both ion modes, the four groups exhibited similar major peak distributions across the retention time range, but several peaks differed clearly in intensity, indicating that drying affected the composition and abundance of non-volatile compounds. A total of 148 compounds were identified or tentatively characterized (Table S3) based on accurate mass data, MS/MS fragmentation patterns, and comparison with authentic standard library, databases and published literature [26]. According to the MSI annotation criteria, 14 compounds (9.5%) were assigned as Level 1, 122 compounds (82.4%) as Level 2, and 12 compounds (8.1%) as Level 3. Features classified as MSI Level 4 were not included in the annotated compound list, and the MSI level of each compound is provided in Table S3. These compounds mainly included amino acids, organic acids, sugars, phenylethanoid glycosides (PhGs), flavonoids, lignans, iridoids, terpenes, cyclohexyl ethanol derivatives (CEDs), and lipids. PhGs were the most abundant class, followed by organic acids, lignans, flavonoids, and terpenes (Figure 5A), indicating that phenolic compounds and their derivatives constituted a major part of the non-volatile compounds in *Forsythia suspensa* flowers.

#### 3.4.2. Multivariate Analysis of Non-Volatile Compounds

The PCA score plot provided an unsupervised overview of the non-volatile compound profiles across the four drying treatments. The QC samples clustered tightly, indicating good analytical stability and repeatability of the LC-MS analysis (Figure 5B). The samples from different drying treatments were distributed differently in the PCA score plot. FD samples were separated from the other treatments along PC1, whereas HAD and ID samples were located relatively close to each other. SD samples showed an intermediate distribution pattern. These distribution patterns suggested that drying methods contributed to differences in the non-volatile compound profiles.

PLS-DA was further used as a supervised exploratory analysis to visualize group separation trends and generate VIP values for differential compound screening. The PLS-DA score plot showed different distribution patterns across the four drying treatments (Figure 5C). Based on VIP > 1 and q-value < 0.05, 62 non-volatile compounds were screened as differential compounds across the four drying treatments, and detailed information is provided in Table S4. The permutation test showed R^2^ and Q^2^ intercepts of 0.299 and −0.569, respectively, indicating that the model did not exhibit obvious overfitting (Figure 5D). The heatmap of these compounds showed a relatively distinct abundance pattern in HAD, whereas FD, ID, and SD were more similar to one another (Figure S3C). Among these differential compounds, HAD showed higher relative abundances of several PhGs, lignans, and iridoids, including forsythoside D, cornoside, rengyoside B, pinoresinol, forsythenside A, forsythenside B, benzoylated iridoid glycoside, and forsythoside M. By contrast, FD showed higher relative abundances of aloin B, gardoside, malic acid, succinic acid, and hydroxybenzoic acid, whereas ID showed higher relative levels of pyroglutamic acid, plantainoside A, forsythialanside E, forsythoside I isomer, and betulinic acid. SD was characterized by higher relative abundances of several organic acids together with a few phenolic compounds.

**Figure 6:**
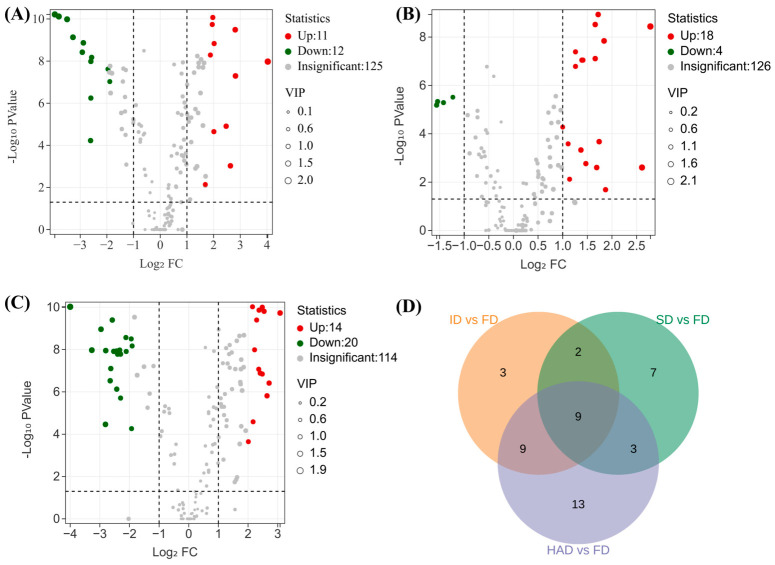
Volcano plots and a Venn diagram of differential compounds in *Forsythia suspensa* flowers subjected to different drying methods. (**A**) FD vs. ID; (**B**) FD vs. SD; (**C**) FD vs. HAD; (**D**) Venn diagram of differential compounds identified in the three comparison groups. Differential compounds were screened using the criteria VIP > 1.3, q-value < 0.05, and |log_2_FC| > 1. FD, freeze-drying; ID, indoor shade drying; SD, sun drying; HAD, hot-air drying.

FD was used as the reference because freeze-drying is generally regarded as a mild dehydration method that better preserves heat sensitive and bioactive constituents than thermal drying treatments, making it an appropriate reference for evaluating the effects of the other drying methods [13,38,39]. Based on VIP > 1.3, q-value < 0.05, and |log_2_FC| > 1, 23, 21, and 34 differential compounds were identified in ID, SD, and HAD, respectively, relative to FD. HAD showed the greatest compositional change among the three drying methods (Figure 6A–C). These differential compounds were mainly distributed among phenylethanoid glycosides, lignans, iridoids, organic acids, terpenes, and flavonoids (Table 2). In addition, nine differential compounds were shared among the three pairwise comparisons (Figure 6D). Because these shared compounds were consistently altered across all comparisons, they were considered common and stable differential markers induced by the drying treatments and were therefore used for subsequent correlation analysis with the electronic tongue data. Overall, the drying method markedly affected the chemical composition of *Forsythia suspensa* flowers.

### 3.5. Correlation Analysis of Aroma and Taste with Chemical Compounds

The E-nose and E-tongue results in Figure 1 showed clear differences between the four drying methods, while HS-GC-MS and LC-MS further revealed distinct changes in volatile and non-volatile compounds. Spearman correlation analysis was used to examine the relationships between the E-nose and E-tongue variables and the corresponding compounds.

#### 3.5.1. Correlation Between E-Nose Responses and Aroma-Active Compounds

**Figure 7:**
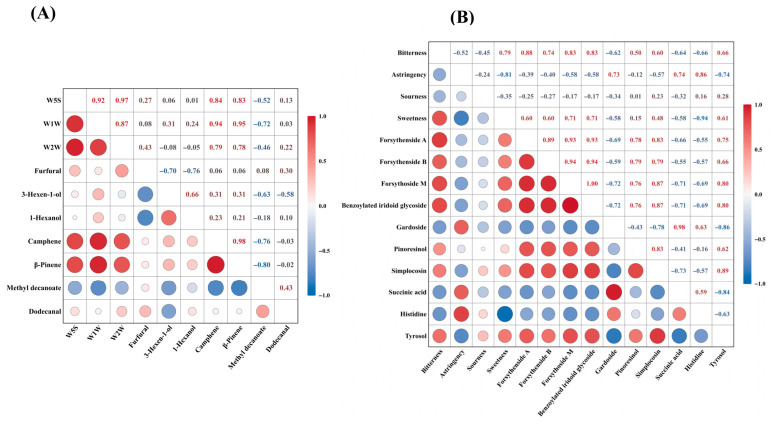
Correlation analysis between E-nose sensor responses and aroma-active compounds (**A**), and between E-tongue attributes and differential non-volatile compounds (**B**) in *Forsythia suspensa* flowers subjected to different drying methods. Circle color and size represent Spearman’s correlation coefficients, with red and blue indicating positive and negative correlations, respectively.

The Spearman correlation analysis of 17 differential volatile compounds (VIP > 1, q-value < 0.05) showed distinct correlation patterns with all E-nose sensors (Figure S5). Stronger positive correlations were mainly observed for a small group of compounds, whereas the remaining compounds showed weaker or opposite correlations [40]. The correlations between W5S, W1W, and W2W and seven key aroma-active compounds identified by HS-GC-MS are shown in Figure 7A. Among these compounds, camphene and β-pinene showed the strongest positive correlations with all three sensors. The correlation coefficients with W5S were 0.84 and 0.83, respectively, whereas those with W1W reached 0.94 and 0.95. Positive correlations were also observed with W2W, with coefficients of 0.79 and 0.78. Overall, camphene and β-pinene were more strongly associated with the dominant E-nose sensor responses than the other selected compounds.

By contrast, methyl decanoate showed negative correlations with W5S, W1W, and W2W, with coefficients of −0.52, −0.72, and −0.46, respectively, whereas dodecanal was only weakly correlated with these sensors. Furfural was negatively correlated with 3-hexen-1-ol and 1-hexanol, while 3-hexen-1-ol and 1-hexanol were positively correlated. Overall, camphene and β-pinene were most closely associated with the dominant E-nose sensor responses, whereas methyl decanoate, dodecanal, furfural, 3-hexen-1-ol, and 1-hexanol showed distinct correlation patterns.

#### 3.5.2. Correlation Between E-Tongue Responses and Differential Compounds

A total of 46 differential compounds were obtained after merging and deduplication of the compounds identified from the three pairwise comparisons of FD versus SD, ID, and HAD based on VIP > 1.3, q-value < 0.05, and |log2FC| ≥ 1. Spearman correlation analysis of these compounds revealed distinct correlation patterns with all E-tongue attributes (Figure S6). The clearest correlations were observed for bitterness and aftertaste-B, whereas correlations with saltiness, richness, and umami were generally weaker, and sweetness showed the opposite trend [41,42].

The correlations between the selected E-tongue attributes and nine common differential compounds identified in all three comparisons are shown in Figure 7B. Among these compounds, forsythenside A, forsythenside B, forsythoside M, and benzoylated iridoid glycoside showed the strongest positive correlations with bitterness and aftertaste-B, indicating that they were most closely associated with the bitter characteristics of *Forsythia suspensa* flowers after drying [20]. Pinoresinol and simplocosin also showed positive correlations with bitterness and aftertaste-B, although these relationships were weaker. By contrast, gardoside, succinic acid, histidine, and tyrosol showed weaker or less consistent relationships with the main E-tongue attributes. In addition, sweetness showed the opposite trend for several compounds, suggesting that stronger bitter responses were accompanied by weaker sweet perception.

#### 3.5.3. Integrated Analysis of Flavor Differences Among Drying Methods

Camphene and β-pinene were more closely associated with W5S, W1W, and W2W, whereas forsythenside A, forsythenside B, forsythoside M, and benzoylated iridoid glycoside were more strongly associated with bitterness and aftertaste-B. These findings suggest that variations in aroma were primarily related to volatile terpenes, whereas differences in taste were mainly associated with non-volatile phenylethanoid glycosides and iridoid glycosides [20].

From a practical perspective, FD was associated with a relatively mild taste profile in the present study. However, previous studies have suggested that freeze-drying generally requires higher equipment investment and is less suitable for large-scale production than conventional thermal drying methods [43]. ID and SD provide simpler alternatives, although their lower controllability may affect processing consistency. By contrast, HAD offers better operational control and has greater potential for standardized large-scale production [44]. Therefore, HAD may represent a more practical processing option for *Forsythia suspensa* flowers, provided that the drying conditions are optimized to preserve aroma quality and maintain acceptable taste characteristics.

### 3.6. Sensory Evaluation

**Figure 8:**
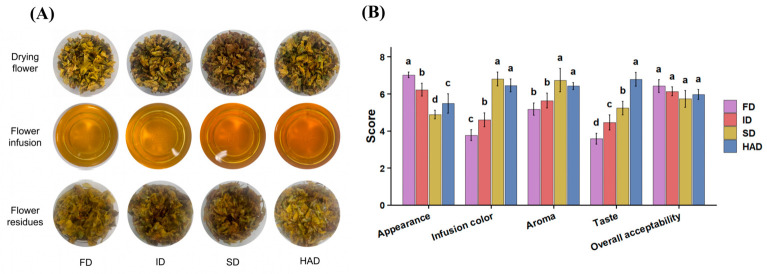
(**A**) Appearance of dried *Forsythia suspensa* flowers, infusions, and residues under different drying treatments. (**B**) Sensory scores of the infusions under different drying treatments. Different letters above the bars indicate significant differences among treatments (*p* < 0.05). FD, freeze-drying; ID, indoor shade drying; SD, sun drying; HAD, hot-air drying.

To examine whether the flavor differences revealed by instrumental and chemical analyses were also reflected in sensory perception, a preliminary sensory evaluation was subsequently conducted to provide supplementary sensory information. Visual comparison showed that the appearance of the dried flowers, infusions, and residues differed across the four drying treatments (Figure 8A). The infusion prepared from FD samples was lighter and brighter, whereas the infusions from SD and HAD samples showed deeper yellow-brown coloration, with ID showing an intermediate appearance. The infusion colors of SD and HAD were relatively similar. The sensory attributes also varied across the drying treatments. FD received higher appearance scores, whereas SD and HAD were associated with higher scores for infusion color and aroma. HAD was also characterized by stronger taste intensity, while differences in overall acceptability were less pronounced (Figure 8B and Table S5).

The preliminary sensory evaluation results were generally in agreement with the instrumental and chemical analyses and provided supplementary information for interpreting the effects of drying treatments. FD appeared to better preserve visual quality, whereas SD and HAD were associated with stronger infusion color and aroma intensity. HAD, in particular, showed stronger taste intensity. Given the limited number of participants, these sensory results should be interpreted as supportive information rather than as a comprehensive descriptive sensory characterization.

## 4. Conclusions

Different drying methods significantly affected the flavor characteristics of *Forsythia suspensa* flowers. Electronic nose (E-nose) and electronic tongue (E-tongue) analyses showed different aroma and taste response patterns across samples prepared by freeze-drying (FD), indoor shade drying (ID), sun drying (SD), and hot-air drying (HAD). HS-GC-MS analysis showed that aldehydes were the major volatile class, while the composition and odor contribution of volatile compounds differed across drying treatments. SD was characterized by green, fatty, and citrus-like odor notes mainly linked to aldehydes. HAD showed pine-like, woody, and camphor-like notes associated with terpenes, whereas ID was associated with herbal and floral notes.

LC-MS analysis showed that drying changed the non-volatile profile, particularly among phenylethanoid glycosides, lignans, iridoids, organic acids, and flavonoids. HAD was associated with a distinct non-volatile pattern and with compounds contributing to bitterness and aftertaste. Correlation analysis suggested that terpenes were more closely linked to aroma variation, whereas phenylethanoid glycosides and iridoid glycosides were more closely linked to taste differences. The preliminary sensory evaluation provided supplementary information for these instrumental findings: FD was favorable for maintaining a lighter infusion appearance, whereas HAD showed higher taste intensity scores and, together with SD, higher infusion color and aroma scores.

Overall, these findings indicate that the choice of drying method for *Forsythia suspensa* flowers should depend on the desired flavor characteristics and chemical profile. FD may be preferred when appearance preservation is prioritized, whereas HAD may be more suitable when higher aroma and taste scores, together with overall acceptability, are desired. These results provide a practical basis for drying method selection, with HAD showing greater potential for standardized processing under the conditions of this study.

## Figures and Tables

**Table 1:** Odor activity values of aroma-active volatile compounds (OAV > 1) in *Forsythia suspensa* flowers under different drying methods.

Compound ID	Compounds Name	OT (μg/g)	Odor Activity Values				Odor Description
			FD	ID	SD	HAD	
Aldehydes (14)							
VOC5	Hexanal	0.0045	406.61 ± 69.77 ^b^	277.25 ± 52.28 ^c^	608.51 ± 80.95 ^a^	532.15 ± 12.60 ^a^	Grassy, green, tallowy
VOC9	Heptanal	0.0028	846.67 ± 134.74 ^a^	225.69 ± 38.88 ^b^	976.49 ± 143.59 ^a^	405.66 ± 8.10 ^b^	Fatty, green, citrus
VOC16	Benzaldehyde	0.3500	1.89 ± 0.29 ^c^	1.80 ± 0.30 ^c^	2.58 ± 0.24 ^b^	4.84 ± 0.41 ^a^	Almond-like, sweet, cherry, nutty
VOC23	Octanal	0.0069	891.07 ± 187.47 ^a^	363.05 ± 62.47 ^b^	1027.42 ± 155.86 ^a^	843.92 ± 114.25 ^a^	Citrus, fatty, green, fresh
VOC30	Benzeneacetaldehyde	0.0040	81.36 ± 17.11 ^c^	206.95 ± 29.87 ^b^	137.43 ± 17.02 ^c^	499.32 ± 59.61 ^a^	Nutty, sweet, roasted, baked
VOC34	Nonanal	0.0011	11,080.70 ± 2302.43 ^b^	4818.32 ± 816.07 ^c^	14,736.05 ± 1927.92 ^a^	10,910.83 ± 1038.68 ^b^	Sweet, woody
VOC39	4-Ethylbenzaldehyde	0.0400	4.21 ± 0.86 ^a^	5.61 ± 2.87 ^a^	7.19 ± 0.34 ^a^	6.14 ± 2.71 ^a^	Woody, spicy
VOC43	Safranal	0.0030	65.02 ± 13.95 ^c^	109.53 ± 17.12 ^b^	80.71 ± 17.99 ^bc^	268.71 ± 33.47 ^a^	Fat, soap, lemon, green
VOC44	Decanal	0.0030	728.74 ± 156.14 ^a^	139.27 ± 22.44 ^b^	756.87 ± 99.38 ^a^	305.50 ± 22.91 ^b^	Citrus, woody, spicy
VOC45	β-Cyclocitral	0.0030	22.52 ± 8.72 ^c^	40.20 ± 10.26 ^b^	42.58 ± 4.84 ^b^	99.74 ± 9.27 ^a^	Citrus, green, herbal
VOC49	Undecanal	0.0125	484.00 ± 108.52 ^b^	116.13 ± 19.82 ^d^	613.39 ± 73.58 ^a^	259.86 ± 20.34 ^c^	Waxy, fatty, citrus, soapy
VOC54	Dodecanal	0.0630	33.49 ± 7.59 ^b^	7.00 ± 1.28 ^d^	54.03 ± 5.68 ^a^	21.86 ± 2.56 ^c^	Floral, fatty, green, lemon-like
VOC56	Tridecanal	0.0700	11.49 ± 2.71 ^a^	1.94 ± 0.25 ^b^	10.41 ± 1.12 ^a^	4.58 ± 1.17 ^b^	Waxy, fatty, soapy
VOC58	Tetradecanal	0.0640	36.64 ± 8.38 ^a^	7.73 ± 1.31 ^b^	41.48 ± 3.56 ^a^	16.26 ± 1.62 ^b^	Woody, spicy, medicinal, powdery, herbal
**Esters (6)**							
VOC12	Methyl hexanoate	0.0700	11.41 ± 2.59 ^a^	14.10 ± 7.51 ^a^	14.90 ± 3.97 ^a^	15.09 ± 1.30 ^a^	Fruity, pineapple-like, sweet
VOC26	Methyl heptanoate	0.0040	322.68 ± 11.66 ^a^	-	156.91 ± 46.42 ^b^	-	Sweet, bready, caramel
VOC36	Methyl octylate	0.2000	5.55 ± 1.76 ^a^	3.08 ± 2.31 ^ab^	3.64 ± 2.27 ^ab^	1.26 ± 0.31 ^b^	Almond, cherry, sweet
VOC48	Ethyl nonanoate	0.0193	13.49 ± 3.37 ^b^	8.45 ± 1.80 ^c^	18.46 ± 2.05 ^a^	18.89 ± 2.11 ^a^	Camphor, mint, green
VOC51	Methyl decanoate	0.0088	31.03 ± 3.38 ^a^	16.00 ± 9.06 ^bc^	22.32 ± 7.18 ^ab^	5.73 ± 3.04 ^c^	Citrus, lemon-like, woody, spicy, juicy
VOC57	Methyl laurate	0.0260	12.80 ± 3.69 ^a^	10.22 ± 5.36 ^a^	10.04 ± 4.82 ^a^	12.31 ± 0.87 ^a^	Fatty, fruity, waxy
**Terpenes (9)**							
VOC13	α-Thujene	0.0010	-	51.58 ± 16.47 ^a^	57.04 ± 14.28 ^a^	-	Green, herbal, woody, resinous
VOC14	α-Pinene	0.1200	12.95 ± 2.20 ^c^	20.21 ± 3.35 ^b^	25.77 ± 2.95 ^b^	59.67 ± 3.81 ^a^	Terpenes
VOC15	Camphene	0.0150	6.28 ± 1.59 ^c^	15.68 ± 2.09 ^bc^	18.36 ± 1.79 ^b^	34.00 ± 9.48 ^a^	Waxy, fatty, oily
VOC18	Sabinene	0.9800	2.48 ± 0.44 ^c^	3.34 ± 0.52 ^bc^	4.20 ± 0.53 ^b^	8.12 ± 0.72 ^a^	Waxy, oily, fatty
VOC19	β-Pinene	0.1400	34.45 ± 6.77 ^c^	54.41 ± 8.82 ^b^	72.11 ± 7.80 ^b^	156.72 ± 13.66 ^a^	Pine-like, woody, green, resinous
VOC22	β-Myrcene	0.0150	44.97 ± 7.69 ^c^	66.41 ± 11.66 ^b^	83.81 ± 8.24 ^b^	126.00 ± 11.63 ^a^	Herbal, green, balsamic, resinous
VOC24	α-terpinene	0.0850	3.34 ± 0.74 ^b^	1.92 ± 0.58 ^c^	6.02 ± 0.56 ^a^	5.57 ± 0.25 ^a^	Citrus, fresh, lemon-like, herbal
VOC25	p-Cymene	0.0133	12.46 ± 4.61 ^c^	15.92 ± 3.32 ^bc^	21.65 ± 3.99 ^b^	39.99 ± 2.85 ^a^	Grassy, green, fatty, herbaceous
VOC31	γ-Terpinene	0.0550	4.32 ± 0.65 ^c^	4.84 ± 0.77 ^c^	7.81 ± 0.93 ^b^	10.04 ± 0.86 ^a^	Nutty, roasted, cocoa-like
**Alcohols (6)**							
VOC2	1-Pentanol	0.1503	-	1.10 ± 0.25 ^a^	-	-	Alcoholic, fatty, green
VOC7	3-Hexen-1-ol	0.0700	6.53 ± 0.71 ^c^	27.61 ± 3.72 ^a^	12.99 ± 1.97 ^b^	13.46 ± 1.22 ^b^	Spicy, herbal, green
VOC8	1-Hexanol	0.1000	4.32 ± 0.47 ^b^	6.24 ± 0.92 ^a^	6.77 ± 0.48 ^a^	4.72 ± 0.30 ^b^	Herbal, woody
VOC20	1-Octen-3-ol	0.0010	120.37 ± 19.57 ^a^	68.73 ± 11.45 ^a^	122.24 ± 28.31 ^a^	108.05 ± 47.22 ^a^	Mushroom-like, earthy, green
VOC35	Phenylethyl Alcohol	0.3900	1.97 ± 0.37 ^b^	5.91 ± 0.85 ^a^	2.76 ± 0.35 ^b^	5.82 ± 0.21 ^a^	Camphor, fatty
VOC50	4-Hydroxy-3-methoxystyrene	0.0120	10.49 ± 2.42 ^b^	11.57 ± 2.21 ^b^	12.11 ± 0.76 ^b^	25.36 ± 2.40 ^a^	Floral, rose, cherry-like
**Heterocyclics (2)**							
VOC6	Furfural	0.7700	1.23 ± 0.15 ^a^	0.201 ± 0.077 ^c^	0.885 ± 0.039 ^b^	1.18 ± 0.04 ^a^	Sweet, almond-like, caramel
VOC17	5-Methyl-2-furancarboxaldehyde	0.5000	0.483 ± 0.075 ^c^	0.707 ± 0.123 ^b^	0.535 ± 0.028 ^c^	1.40 ± 0.10 ^a^	Oily, fatty, woody
**Ethers (1)**							
VOC28	Cineole	0.0013	173.39 ± 55.72 ^b^	155.10 ± 33.18 ^b^	235.47 ± 42.92 ^ab^	302.76 ± 28.97 ^a^	Camphor, minty, green

Note: OT, odor threshold in aqueous solution; OAV, odor activity value. FD, freeze drying; ID, infrared drying; SD, sun drying; HAD, hot-air drying. Bold text indicates compound classes, and numbers in parentheses indicate compound counts. OAV was calculated as the concentration of each compound divided by its corresponding odor threshold. Values are expressed as mean ± standard deviation. Different superscript letters within the same row indicate significant differences between different drying methods according to Duncan’s multiple range test (*p* < 0.05). Odor threshold values and odor descriptions were obtained from published literature [30,31,32].

**Table 2:** Differential compounds in the ID, SD, and HAD groups compared with the FD group based on OPLS-DA (VIP > 1.3, q-value < 0.05, and |log2FC| ≥ 1).

Compound ID	Compounds Name	Class	log2FC (ID/FD)	log2FC (SD/FD)	log2FC (HAD/FD)
comp2	Histidine	Amino acid	−2.61	\	−2.81
comp3	Arginine	Amino acid	−1.96	\	\
comp8	Sorbitol	Sugars	\	\	−2.11
comp14	Malic acid	Organic acid	\	\	−2.12
comp16	Valine	Amino acid	2.82	\	2.48
comp22	Succinic acid	Organic acid	−3.95	\	−1.94
comp23	2-(3,4-Dihydroxyphenyl) ethyl *β*-D-glucopyranoside	PhGs	\	\	2.22
comp27	Cornoside	PhGs	\	\	2.36
comp28	Tyrosol	PhGs	4.03	\	2.71
comp29	Rengyoside B	CEDs	\	\	2.37
comp36	trans-3-Indoleacrylic acid	Others	−2.6	\	−2.3
comp39	5′-S-Methyl-5′-thioadenosine	Others	−3.49	−1.23	−2.58
comp41	Salidroside	PhGs	−2.57	\	−2.96
comp47	Hydroxytyrosol apiosylglucoside	PhGs	−2.6	\	−2.43
comp50	Gardoside	Iridoids	−3.27	−1.54	−2.4
comp51	Forsythenside B	CEDs	2.02	1.84	2.54
comp57	Geniposide isomer	Iridoids	−2.92	\	−2.53
comp63	p-Coumaroylquinic acid I	Organic acid	\	1.26	\
comp64	Sinapic acid glucoside isomer	Organic acid	−1.89	\	\
comp65	Asperuloside-type iridoid glycoside	Iridoids	\	1.39	\
comp66	Suspenoidside E	Iridoids	\	1.37	\
comp68	Forsythenside A	CEDs	2.01	1.74	2.64
comp77	Forsythoside M	PhGs	1.88	1.66	2.29
comp79	Benzoylated iridoid glycoside	Iridoids	1.95	1.66	2.48
comp85	Caffeoyl phenylethanoid glycoside isomer II	PhGs	\	1	\
comp88	Plantamajoside	PhGs	\	1.69	2.01
comp97	Forsyoxaside F	PhGs	1.69	\	\
comp101	Adoxosidic acid-6′-oleuroperic ester	Iridoids	2.63	2.61	\
comp109	Swertiamacroside	PhGs	\	\	−3.27
comp114	Campneoside I	PhGs	2.47	1.47	\
comp118	Aloin B	Flavonoids	−2.88	−1.42	−2.3
comp119	Phillygenin	Lignans	\	1.26	\
comp121	Simplocosin	Lignans	1.97	1.72	2.15
comp123	Acanthoside B	Lignans	\	1.41	\
comp124	Pinoresinol	Lignans	2.81	2.78	3.08
comp126	Forsythenside L	CEDs	−3.8	\	−4
comp127	Forsypensin C	Terpenes	\	−1.56	−2.8
comp129	Forsypensin E	Terpenes	\	\	−2.43
comp130	Wilforlide B	Terpenes	\	\	−1.91
comp133	Eicosapentaenoic acid	Organic acid	\	1.87	\
comp135	18-*β*-Glycyrrhetinic acid	Terpenes	\	1.14	2.17
comp141	betulonic acid	Terpenes	\	\	−2.33
comp142	Rubicoumaric acid	Terpenes	\	\	−2.65
comp144	Betulinic acid	Terpenes	\	\	−1.93
comp145	Olean-12-ene-3,11-dione	Terpenes	\	\	−2.63
comp146	*β*-Amyrone	Terpenes	\	\	2.41

Note: PhGs, phenylethanoid glycosides; CEDs, cyclohexyl ethanol derivatives. “\” indicates that the compound was not significantly changed in the corresponding pairwise comparison.

## Data Availability

The original contributions presented in this study are included in the article/Supplementary Material. Further inquiries can be directed to the corresponding authors.

## Supplementary Materials

The following supporting information can be downloaded at: https://www.mdpi.com/article/10.3390/foods15101757/s1, Figure S1: PLS-DA and VIP analyses of electronic nose and electronic tongue of *Forsythia suspensa* flowers under different drying methods. (A) PLS-DA score plot of electronic nose; (B) VIP analysis of electronic nose variables; (C) PLS-DA score plot of electronic tongue; (D) VIP analysis of electronic tongue variables; Figure S2. (A) HS-GC-MS total ion chromatograms (TICs) of volatile compounds in Forsythia suspensa flowers under different drying methods. (B) Clustering heatmap of the 17 differential volatile compounds screened based on VIP > 1 and q-value < 0.05 across the four drying treatments. FD, freeze-drying; ID, indoor shade drying; SD, sun drying; HAD, hot-air drying; Figure S3. LC-MS total ion chromatograms (TICs) and clustering heatmap of differential non-volatile compounds in Forsythia suspensa flowers under different drying methods. (A) TICs in positive ion mode (ESI+). (B) TICs in negative ion mode (ESI−). (C) Hierarchical clustering heatmap of the 62 differential non-volatile compounds screened based on VIP > 1 and q-value < 0.05. FD, freeze-drying; ID, indoor shade drying; SD, sun drying; HAD, hot-air drying; Figure S4. OPLS-DA analysis of non-volatile compounds based on LC-MS analysis in *Forsythia suspensa* flowers under different drying methods with ID, SD, and HAD each compared with FD: (A) OPLS-DA score plot and (B) 200 permutation test of FD vs. ID; (C) OPLS-DA score plot and (D) 200 permutation test of FD vs. SD; (E) OPLS-DA score plot and 200 permutation test of FD vs. HAD.; Figure S5. Spearman correlation analysis between E-nose sensors and differential volatile compounds in *Forsythia suspensa* flowers under different drying methods. Differential volatile compounds were selected from the HS-GC-MS data based on VIP > 1 and q-value < 0.05; Figure S6. Spearman correlation analysis between E-tongue attributes and differential non-volatile compounds in *Forsythia suspensa* flowers under different drying methods. Differential compounds were obtained from the merged results of the three pairwise comparisons of FD versus SD, ID, and HAD after duplicate removal, based on VIP > 1.3, q-value < 0.05, and |log2FC| ≥ 1; Table S1. Gradient elution program for LC–MS analysis; Table S2. Volatile compounds identified in *Forsythia suspensa* flowers under different drying methods by HS-GC-MS; Table S3. Identification of non-volatile metabolites in *Forsythia suspensa* flowers by LC–MS; Table S4. Differential volatile and non-volatile compounds screened based on VIP > 1 and q-value < 0.05 across the four drying treatments; Table S5. Sensory evaluation scores of *Forsythia suspensa* flower infusions under different drying treatments.
