# Supplementary material for: Effects of Different Drying Methods on the Flavor Characteristics and Chemical Profile of Forsythia suspensa Flowers Using Electronic Sensors and Mass Spectrometry
Source: Foods. 2026 May 15;15(10):1757. doi: 10.3390/foods15101757 (PMC13205628; doi:10.3390/foods15101757)
Supplement: Supplementary file 1 [file foods-15-01757-s001.zip › foods-4298773-supplementary.pdf]

Effects of Different Drying Methods on the Flavor Characteristics and Chemical Profile *of Forsythia suspensa* Flowers Using Electronic Sensors and Mass Spectrometry

Qingling Xie, Jiangyi Luo, Ling Liang, Wei Su, Mengying Lyu, Caiyun Peng, Bin Li, Wei Wang*, Hanwen Yuan*

TCM and Ethnomedicine Innovation & Development International Laboratory, School of Pharmacy, Hunan University of Chinese Medicine, Changsha, 410208, China; xieql12@126.com (Q.X.); jyiluo1998@163.com (J.L.); lliang901@stu.hnucm.edu.cn (L.L.); suwei0310@163.com (S.W.); dancytime@163.com (M.L.); 002142@hnucm.edu.cn (C.P.); libin@hnucm.edu.cn (B.L.).

* Correspondence: wangwei402@hotmail.com (W. W), Hanwyuan@hnucm.edu.cn (H. Y).

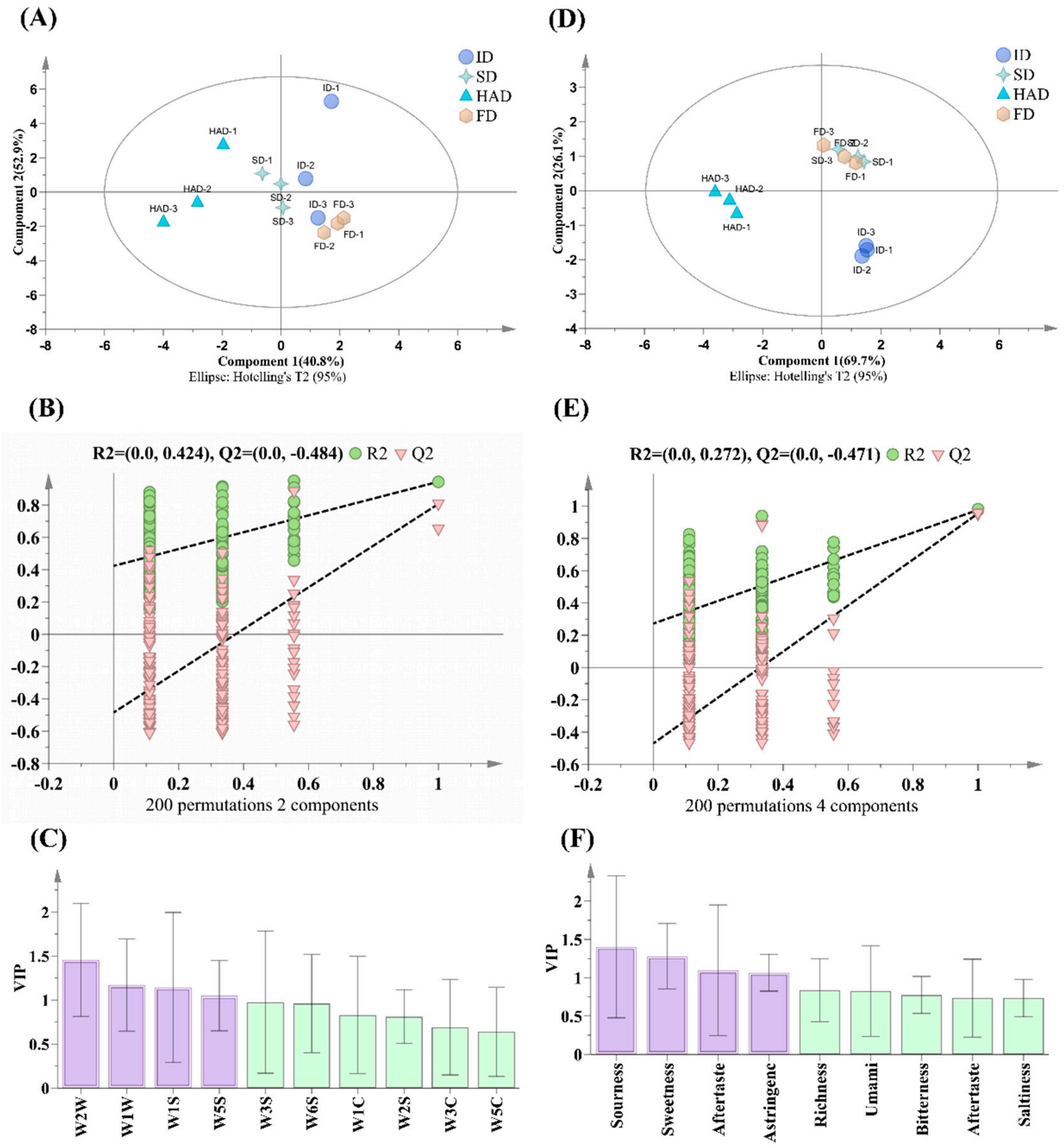

Figure S1. PLS-DA and VIP analyses of electronic nose and electronic tongue of *Forsythia suspensa* flowers under different drying methods. (A) PLS-DA score plot of electronic nose; (B) VIP analysis of electronic nose variables; (C) PLS-DA score plot of electronic tongue; (D) VIP analysis of electronic tongue variables. ID, indoor shade drying. SD, sun drying. HAD, hot-air drying. FD, freeze-drying. Aftertaste-A indicates astringent aftertaste, and Aftertaste-B indicates bitter aftertaste. Purple bars indicate VIP > 1.

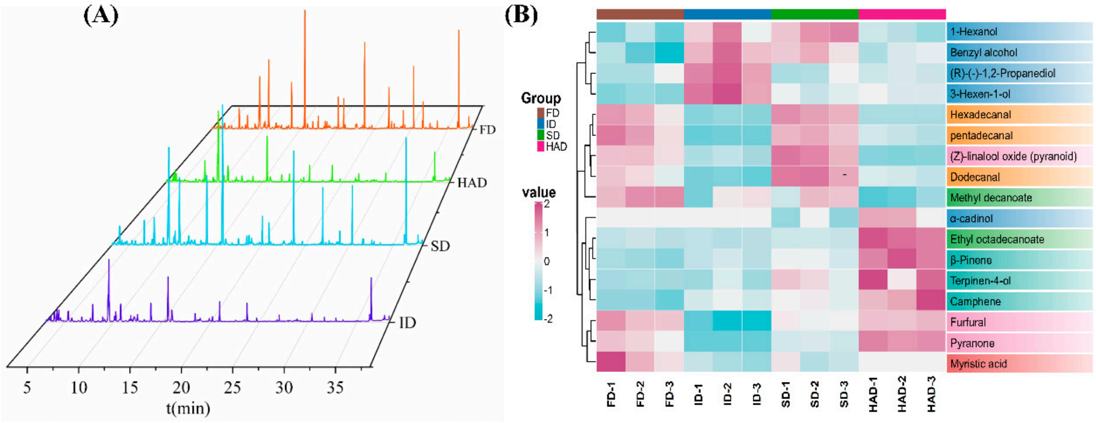

Figure S2. (A) HS-GC-MS total ion chromatograms (TICs) of volatile compounds in *Forsythia suspensa* flowers under different drying methods. (B) Clustering heatmap of the 17 differential volatile compounds screened based on VIP > 1 and q-value < 0.05 across the four drying treatments. FD, freeze-drying; ID, indoor shade drying; SD, sun drying; HAD, hot-air drying.

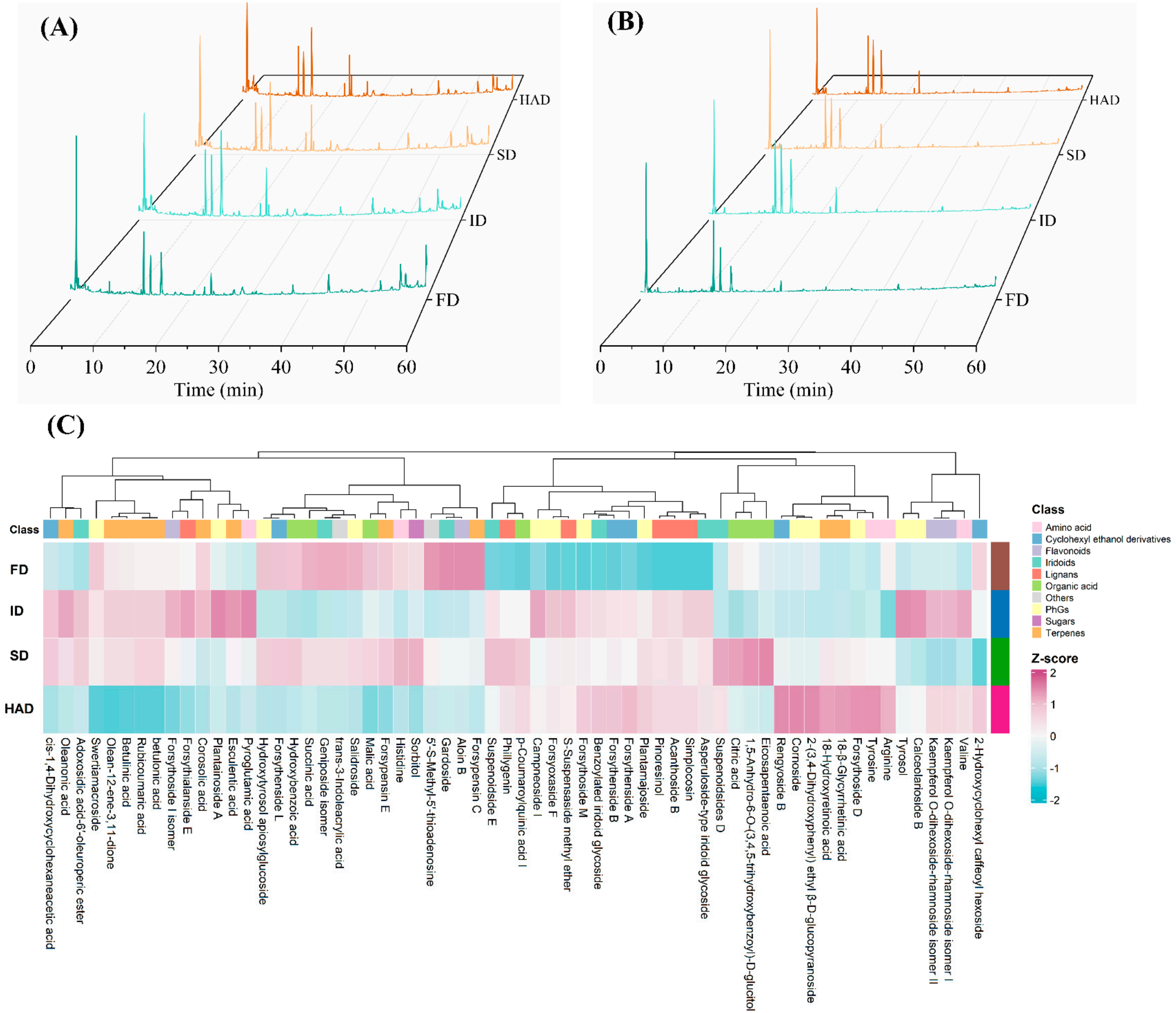
(A)
HAD
SD
ID
FD
0
10
20
30
40
50
60
Time (min)
(B)
HAD
SD
ID
FD
0
10
20
30
40
50
60
Time (min)
(C)
Class
FD
ID
SD
HAD
cis-1,4-Dihydroxycyclohexaneacetic acid
Oleanonic acid
Adoxosidic acid-6'-oleuroperic ester
Swertiamacroside
Olean-12-ene-3,11-dione
Betulinic acid
Rubicoumaric acid
betulonic acid
Forsythoside I isomer
Forsythialanside E
Corosolic acid
Plantainoside A
Esculentic acid
Pyroglutamic acid
Hydroxytyrosol apiosylglucoside
Forsythenside L
Hydroxybenzoic acid
Succinic acid
Geniposide isomer
trans-3-Indoleacrylic acid
Salidroside
Malic acid
Forsypensin E
Histidine
Sorbitol
5'-S-Methyl-5'-thioadenosine
Gardoside
Aloin B
Forsypensin C
Suspenoidside E
Phillygenin
p-Coumaroylquinic acid I
Campneoside I
Forsyoxaside F
S-Suspensaside methyl ether
Forsythoside M
Benzoylated iridoid glycoside
Forsythenside B
Forsythenside A
Plantamajoside
Pinoresinol
Acanthoside B
Simplocosin
Asperuloside-type iridoid glycoside
Suspenoidsides D
Citric acid
1,5-Anhydro-6-O-(3,4,5-trihydroxybenzoyl)-D-glucitol
Eicosapentaenoic acid
Rengyoside B
Cornoside
2-(3,4-Dihydroxyphenyl) ethyl β-D-glucopyranoside
18-Hydroxyretinoic acid
18-β-Glycyrrhetinic acid
Forsythoside D
Tyrosine
Arginine
Tyrosol
Calceolarioside B
Kaempferol O-dihexoside-rhamnoside isomer I
Kaempferol O-dihexoside-rhamnoside isomer II
Valine
2-Hydroxycyclohexyl caffeoyl hexoside
Class
Amino acid
Cyclohexyl ethanol derivatives
Flavonoids
Iridoids
Lignans
Organic acid
Others
PhGs
Sugars
Terpenes
Z-score
2
1
0
-1
-2

Figure S3. LC-MS total ion chromatograms (TICs) and clustering heatmap of differential non-volatile compounds in *Forsythia suspensa* flowers under different drying methods. (A) TICs in positive ion mode (ESI+). (B) TICs in negative ion mode (ESI−). (C) Hierarchical clustering heatmap of the 62 differential non-volatile compounds screened based on VIP > 1 and q-value < 0.05. FD, freeze-drying; ID, indoor shade drying; SD, sun drying; HAD, hot-air drying.

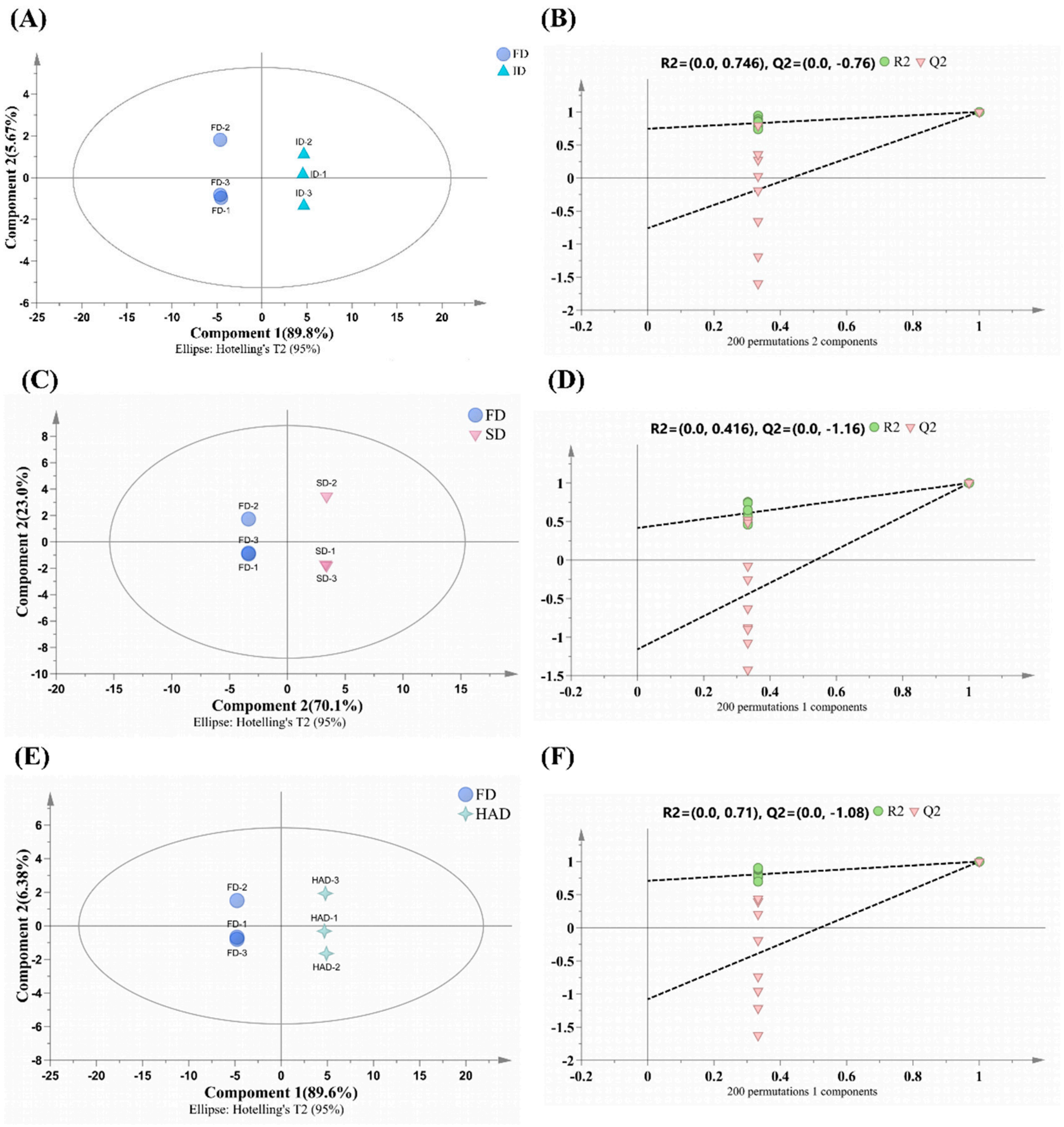

Figure S4. OPLS-DA analysis of non-volatile compounds based on LC-MS analysis in *Forsythia suspensa* flowers under different drying methods with ID, SD, and HAD each compared with FD: (A) OPLS-DA score plot and (B) 200 permutation test of FD vs ID; (C) OPLS-DA score plot and (D) 200 permutation test of FD vs SD; (E) OPLS-DA score plot and 200 permutation test of FD vs HAD. FD, freeze-drying; ID, indoor shade drying; SD, sun drying; HAD, hot-air drying.

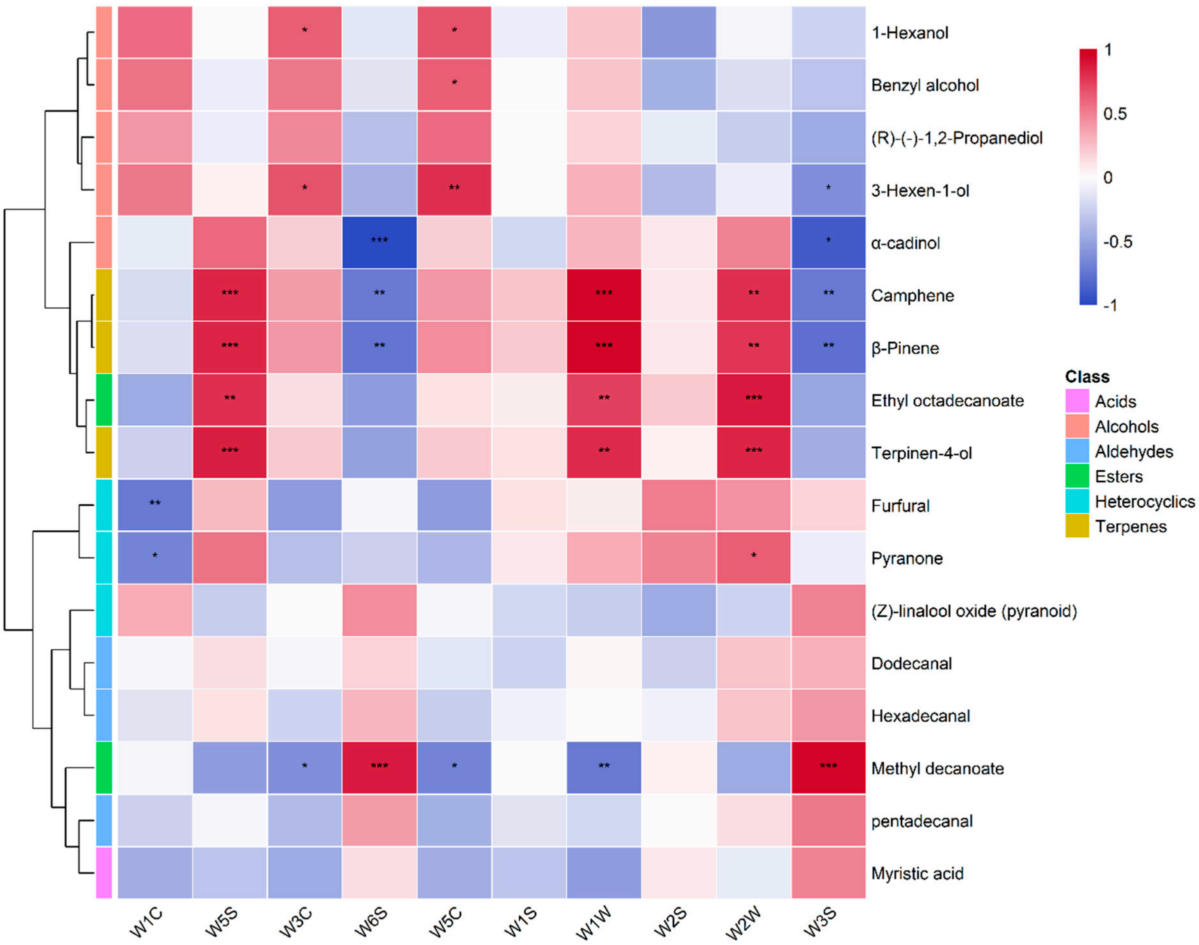

Figure S5. Spearman correlation analysis between E-nose sensors and differential volatile compounds in *Forsythia suspensa* flowers under different drying methods. Differential volatile compounds were selected from the HS-GC-MS data based on VIP > 1 and q-value < 0.05.

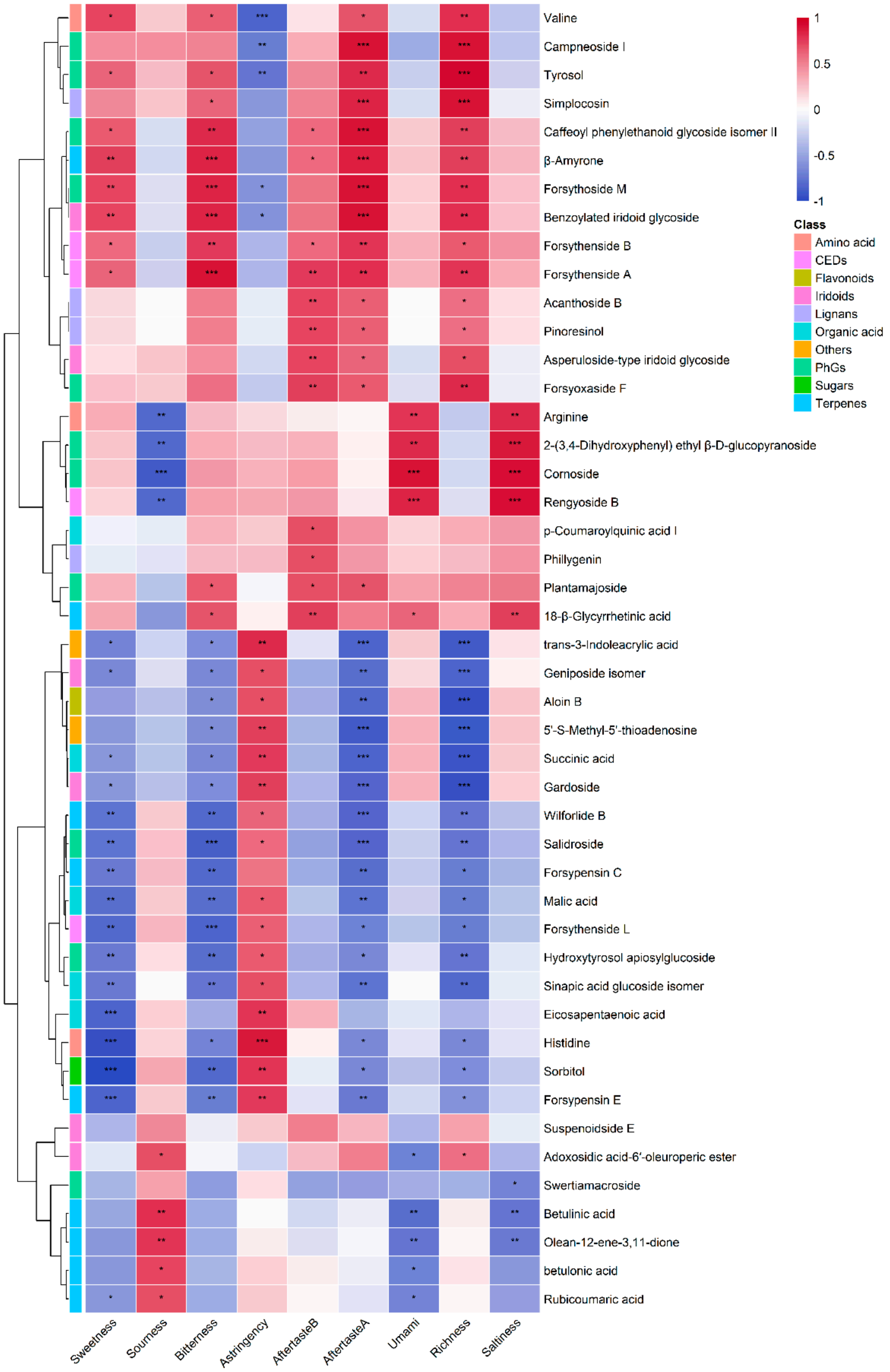

Figure S6. Spearman correlation analysis between E-tongue attributes and differential non-volatile compounds in *Forsythia suspensa* flowers under different drying methods. Differential compounds were obtained from the merged results of the three pairwise comparisons of FD versus SD, ID, and HAD after duplicate removal, based on VIP > 1.3, q-value < 0.05, and |log2FC| ≥ 1.

Table S1. Gradient elution program for LC-MS analysis.

Time (min)	A (%)	B (%)
0	97	3
5	95	5
10	85	15
15	83	17
20	83	17
35	50	50
45	35	65
55	5	95
60	3.5	96.5

Table S2. Volatile compounds identified in *Forsythia suspensa* flowers under different drying methods by HS-GC-MS.

Compound ID	Compounds name	CAS	Formula	RI cal./ RI ref.	Relative content (μg/g)			
					FD	ID	SD	HAD
**Aldehydes (17)**								
VOC5	Hexanal	66-25-1	$C_6H_{12}O$	803/800	1.83 ± 0.31^b	1.25 ± 0.24^c	2.74 ± 0.36^a	2.39 ± 0.06^a
VOC9	Heptanal	111-71-7	$C_7H_{14}O$	904/901	2.37 ± 0.38^a	0.632 ± 0.109^b	2.73 ± 0.40^a	1.14 ± 0.02^b
VOC16	Benzaldehyde	100-52-7	C_7H_6O	963/962	0.663 ± 0.100^c	0.629 ± 0.105^c	0.902 ± 0.084^b	1.70 ± 0.15^a
VOC23	Octanal	124-13-0	$C_8H_{16}O$	1002/1003	6.15 ± 1.29^a	2.51 ± 0.43^b	7.09 ± 1.08^a	5.82 ± 0.79^a
VOC30	Benzeneacetaldehyde	122-78-1	C_8H_8O	1035/1045	0.325 ± 0.068^c	0.828 ± 0.119^b	0.550 ± 0.068^c	2.00 ± 0.24^a
VOC34	Nonanal	124-19-6	$C_9H_{18}O$	1106/1104	12.19 ± 2.53^b	5.30 ± 0.90^c	16.21 ± 2.12^a	12.00 ± 1.14^b
VOC39	4-Ethylbenzaldehyde	4748-78-1	$C_9H_{10}O$	1178/1180	0.168 ± 0.035^a	0.224 ± 0.115^a	0.288 ± 0.013^a	0.246 ± 0.109^a
VOC43	Safranal	116-26-7	$C_{10}H_{14}O$	1198/1201	0.195 ± 0.042^c	0.329 ± 0.051^b	0.242 ± 0.054^{bc}	0.806 ± 0.100^a
VOC44	Decanal	112-31-2	$C_{10}H_{20}O$	1207/1206	2.19 ± 0.47^a	0.418 ± 0.067^b	2.27 ± 0.30^a	0.917 ± 0.069^b
VOC45	β-Cyclocitral	432-25-7	$C_{10}H_{16}O$	1221/1220	0.068 ± 0.026^c	0.121 ± 0.031^b	0.128 ± 0.015^b	0.299 ± 0.028^a
VOC49	Undecanal	112-44-7	$C_{11}H_{22}O$	1308/1307	6.05 ± 1.36^b	1.45 ± 0.25^d	7.67 ± 0.92^a	3.25 ± 0.25^c
VOC53	2,4-Dihydroxy-6-methylbenzaldehyde	487-69-4	$C_8H_8O_3$	1398/1406	-	0.017 ± 0.004^b	0.011 ± 0.003^b	0.073 ± 0.008^a
VOC54	Dodecanal	112-54-9	$C_{12}H_{24}O$	1411/1409	2.11 ± 0.48^b	0.441 ± 0.081^d	3.40 ± 0.36^a	1.38 ± 0.16^c
VOC56	Tridecanal	10486-19-8	$C_{13}H_{26}O$	1513/1512	0.804 ± 0.190^a	0.136 ± 0.018^b	0.729 ± 0.078^a	0.320 ± 0.082^b
VOC58	Tetradecanal	124-25-4	$C_{14}H_{28}O$	1615/1613	2.34 ± 0.54^a	0.495 ± 0.084^b	2.66 ± 0.23^a	1.04 ± 0.10^b
VOC61	Pentadecanal	2765-11-9	$C_{15}H_{30}O$	1717/1715	0.323 ± 0.083^a	0.027 ± 0.004^c	0.301 ± 0.024^a	0.124 ± 0.020^b
VOC65	Hexadecanal	629-80-1	$C_{16}H_{32}O$	1820/1817	0.224 ± 0.060^a	0.024 ± 0.008^b	0.262 ± 0.023^a	0.050 ± 0.001^b
**Esters (14)**								
VOC12	Methyl hexanoate	106-70-7	$C_7H_{14}O_2$	929/925	0.798 ± 0.182^a	0.987 ± 0.526^a	1.04 ± 0.28^a	1.06 ± 0.09^a
VOC26	Methyl heptanoate	106-73-0	$C_8H_{16}O_2$	1025/1023	1.29 ± 0.05^a	-	0.628 ± 0.186^b	-

VOC36	Methyl octylate	111-11-5	$C_9H_{18}O_2$	1132/1126	1.11 ± 0.35[a]	0.617 ± 0.462[ab]	0.729 ± 0.454[ab]	0.252 ± 0.062[b]
VOC48	Ethyl nonanoate	123-29-5	$C_{11}H_{22}O_2$	1300/1296	0.260 ± 0.065[b]	0.163 ± 0.035[c]	0.356 ± 0.040[a]	0.365 ± 0.041[a]
VOC51	Methyl decanoate	110-42-9	$C_{11}H_{22}O_2$	1329/1325	0.273 ± 0.030[a]	0.141 ± 0.080[bc]	0.196 ± 0.063[ab]	0.050 ± 0.027[c]
VOC55	Methyl undecanoate	1731-86-8	$C_{12}H_{24}O_2$	1430/1428	0.687 ± 0.217[a]	0.261 ± 0.204[b]	0.411 ± 0.265[ab]	0.103 ± 0.028[b]
VOC57	Methyl laurate	111-82-0	$C_{13}H_{26}O_2$	1528/1526	0.333 ± 0.096[a]	0.266 ± 0.139[a]	0.261 ± 0.125[a]	0.320 ± 0.023[a]
VOC62	Methyl myristate	124-10-7	$C_{15}H_{30}O_2$	1727/1725	1.99 ± 0.60[a]	2.09 ± 1.59[a]	0.934 ± 0.599[a]	0.359 ± 0.132[a]
VOC64	Ethyl tetradecanoate	124-06-1	$C_{16}H_{32}O_2$	1795/1794	0.056 ± 0.025[b]	0.073 ± 0.014[b]	0.073 ± 0.020[b]	0.180 ± 0.015[a]
VOC67	Methyl hexadecanoate	112-39-0	$C_{17}H_{34}O_2$	1928/1926	0.843 ± 0.292[a]	1.27 ± 1.01[a]	0.520 ± 0.330[a]	0.243 ± 0.086[a]
VOC68	Ethyl hexadecanoate	628-97-7	$C_{18}H_{36}O_2$	1995/1993	0.016 ± 0.001[b]	0.032 ± 0.014[b]	0.025 ± 0.004[b]	0.104 ± 0.013[a]
VOC69	Ethyl linoleate	544-35-4	$C_{20}H_{36}O_2$	2167/2162	0.008 ± 0.003[c]	0.015 ± 0.003[b]	0.011 ± 0.004[bc]	0.036 ± 0.001[a]
VOC70	Ethyl linolenate	1191-41-9	$C_{20}H_{34}O_2$	2174/2169	0.016 ± 0.006[c]	0.030 ± 0.012[bc]	0.033 ± 0.009[b]	0.061 ± 0.004[a]
VOC71	Ethyl octadecanoate	111-61-5	$C_{20}H_{40}O_2$	2195/2195	0.012 ± 0.003[b]	0.010 ± 0.001[b]	0.016 ± 0.001[b]	0.070 ± 0.005[a]
**Terpenes (14)**								
VOC13	α-Thujene	2867-05-2	$C_{10}H_{16}$	930/929	-	0.052 ± 0.016[a]	0.057 ± 0.014[a]	-
VOC14	α-Pinene	80-56-8	$C_{10}H_{16}$	937/937	1.55 ± 0.26[c]	2.43 ± 0.40[b]	3.09 ± 0.35[b]	7.16 ± 0.46[a]
VOC15	Camphene	79-92-5	$C_{10}H_{16}$	951/952	0.094 ± 0.024[c]	0.235 ± 0.031[bc]	0.275 ± 0.027[b]	0.510 ± 0.142[a]
VOC18	Sabinene	3387-41-5	$C_{10}H_{16}$	976/974	2.43 ± 0.43[c]	3.27 ± 0.51[bc]	4.12 ± 0.52[b]	7.95 ± 0.71[a]
VOC19	β-Pinene	127-91-3	$C_{10}H_{16}$	978/979	4.82 ± 0.95[c]	7.62 ± 1.23[b]	10.10 ± 1.09[b]	21.94 ± 1.91[a]
VOC22	β-Myrcene	123-35-3	$C_{10}H_{16}$	993/991	0.675 ± 0.115[c]	0.996 ± 0.175[b]	1.26 ± 0.12[b]	1.89 ± 0.17[a]
VOC24	α-terpinene	99-86-5	$C_{10}H_{16}$	1017/1009	0.284 ± 0.063[b]	0.164 ± 0.049[c]	0.512 ± 0.048[a]	0.473 ± 0.021[a]
VOC25	p-Cymene	99-87-6	$C_{10}H_{14}$	1025/1025	0.166 ± 0.061[c]	0.212 ± 0.044[bc]	0.288 ± 0.053[b]	0.532 ± 0.038[a]
VOC27	(R)-isocarvestrene	1461-27-4	$C_{10}H_{16}$	1029/1027	-	0.940 ± 0.214[b]	0.836 ± 0.284[b]	1.52 ± 0.13[a]
VOC31	γ-Terpinene	99-85-4	$C_{10}H_{16}$	1059/1060	0.238 ± 0.035[c]	0.266 ± 0.043[c]	0.430 ± 0.051[b]	0.552 ± 0.047[a]
VOC40	Terpinen-4-ol	562-74-3	$C_{10}H_{18}O$	1180/1177	0.256 ± 0.007[b]	0.296 ± 0.117[b]	0.593 ± 0.127[ab]	0.909 ± 0.318[a]
VOC42	Carveol	99-48-9	$C_{10}H_{16}O$	1193/1219	0.161 ± 0.029[a]	0.044 ± 0.011[b]	0.185 ± 0.019[a]	0.132 ± 0.110[ab]
VOC47	Thymol	89-83-8	$C_{10}H_{14}O$	1293/1291	0.136 ± 0.029[a]	0.070 ± 0.007[b]	0.164 ± 0.024[a]	0.132 ± 0.019[a]

VOC59	(-)-cubenol	21284-22-0	$C_{15}H_{26}O$	1639/1642	-	0.020 ± 0.008[b]	0.014 ± 0.001[b]	0.040 ± 0.011[a]
**Alcohols (12)**								
VOC1	(±)-2,3-Butanediol	6982-25-8	$C_4H_{10}O_2$	770/773	0.257 ± 0.014[c]	0.541 ± 0.061[b]	0.528 ± 0.057[b]	1.12 ± 0.20[a]
VOC2	1-Pentanol	71-41-0	$C_5H_{12}O$	771/765	-	0.166 ± 0.038[a]	-	-
VOC4	(R)-(-)-1,2-Propanediol	4254-14-2	$C_3H_8O_2$	784/-	0.019 ± 0.000[b]	0.479 ± 0.073[a]	0.024 ± 0.004[b]	0.100 ± 0.018[b]
VOC7	3-Hexen-1-ol	928-96-1	$C_6H_{12}O$	861/857	0.457 ± 0.050[c]	1.93 ± 0.26[a]	0.910 ± 0.138[b]	0.942 ± 0.085[b]
VOC8	1-Hexanol	111-27-3	$C_6H_{14}O$	873/868	0.432 ± 0.047[b]	0.624 ± 0.092[a]	0.677 ± 0.048[a]	0.472 ± 0.030[b]
VOC20	1-Octen-3-ol	3391-86-4	$C_8H_{16}O$	983/980	0.120 ± 0.020[a]	0.069 ± 0.011[a]	0.122 ± 0.028[a]	0.108 ± 0.047[a]
VOC29	Benzyl alcohol	100-51-6	C_7H_8O	1035/1036	0.544 ± 0.216[b]	1.42 ± 0.21[a]	1.23 ± 0.14[a]	0.866 ± 0.120[b]
VOC33	4-Thujanol	17699-16-0	$C_{10}H_{18}O$	1066/1070	0.104 ± 0.022[b]	0.100 ± 0.024[b]	0.182 ± 0.050[a]	0.234 ± 0.016[a]
VOC35	Phenylethyl Alcohol	60-12-8	$C_8H_{10}O$	1115/1116	0.769 ± 0.144[b]	2.31 ± 0.33[a]	1.07 ± 0.14[b]	2.27 ± 0.08[a]
VOC50	4-Hydroxy-3-methoxystyrene	7786-61-0	$C_9H_{10}O_2$	1314/1317	0.126 ± 0.029[b]	0.139 ± 0.027[b]	0.146 ± 0.009[b]	0.305 ± 0.029[a]
VOC52	1-Undecanol	112-42-5	$C_{11}H_{24}O$	1379/1371	0.083 ± 0.009[b]	0.043 ± 0.004[c]	0.090 ± 0.017[ab]	0.109 ± 0.007[a]
VOC60	α-cadinol	481-34-5	$C_{15}H_{26}O$	1653/1653	-	-	0.011 ± 0.001[b]	0.064 ± 0.018[a]
**Heterocyclics (7)**								
VOC6	Furfural	98-01-1	$C_5H_4O_2$	839/833	0.950 ± 0.116[a]	0.155 ± 0.059[c]	0.682 ± 0.030[b]	0.906 ± 0.030[a]
VOC10	Acetylfuran	1192-62-7	$C_6H_6O_2$	915/911	0.433 ± 0.130[b]	0.220 ± 0.022[c]	0.548 ± 0.074[ab]	0.652 ± 0.082[a]
VOC11	2,3-Dimethylpyrazine	5910-89-4	$C_6H_8N_2$	925/926	0.039 ± 0.017[b]	0.076 ± 0.038[ab]	0.104 ± 0.035[ab]	0.153 ± 0.096[a]
VOC17	5-Methyl-2-furancarboxaldehyde	620-02-0	$C_6H_6O_2$	968/965	0.242 ± 0.038[c]	0.354 ± 0.062[b]	0.267 ± 0.014[c]	0.699 ± 0.052[a]
VOC32	2-Acetylpyrrole	1072-83-9	C_6H_7NO	1061/1064	0.138 ± 0.029[a]	0.037 ± 0.008[b]	0.182 ± 0.039[a]	0.171 ± 0.083[a]
VOC37	Pyranone	28564-83-2	$C_6H_8O_4$	1149/1151	1.21 ± 0.24[b]	0.060 ± 0.009[d]	0.718 ± 0.046[c]	1.88 ± 0.08[a]
VOC38	(Z)-linalool oxide (pyranoid)	14009-71-3	$C_{10}H_{18}O_2$	1174/1174	0.696 ± 0.099[b]	0.193 ± 0.063[c]	0.988 ± 0.150[a]	0.035 ± 0.008[c]

**Acids (4)**								
VOC21	Hexanoic acid	142-62-1	$C_6H_{12}O_2$	991/990	0.667 ± 0.158[b]	0.709 ± 0.188[b]	0.815 ± 0.054[b]	1.88 ± 0.15[a]
VOC41	Octanoic acid	124-07-2	$C_8H_{16}O_2$	1183/1180	0.038 ± 0.002[a]	0.016 ± 0.007[a]	0.031 ± 0.031[a]	0.032 ± 0.009[a]
VOC46	Nonanoic acid	112-05-0	$C_9H_{18}O_2$	1275/1273	0.389 ± 0.126[b]	0.221 ± 0.024[b]	0.400 ± 0.060[b]	0.711 ± 0.207[a]
VOC63	Myristic acid	544-63-8	$C_{14}H_{28}O_2$	1760/1768	0.069 ± 0.031[a]	0.011 ± 0.007[b]	0.024 ± 0.015[b]	-
**Ethers (2)**								
VOC3	trans-2,4-dimethyloxetane	29424-94-0	$C_5H_{10}O$	777/-	-	-	-	0.686 ± 0.107[a]
VOC28	Cineole	470-82-6	$C_{10}H_{18}O$	1029/1032	0.225 ± 0.072[b]	0.202 ± 0.043[b]	0.306 ± 0.056[ab]	0.394 ± 0.038[a]
**Ketones (1)**								
VOC66	6,10,14-Trimethyl-2-pentadecanone	502-69-2	$C_{18}H_{36}O$	1849/1844	0.132 ± 0.028[c]	0.274 ± 0.056[b]	0.245 ± 0.011[b]	0.618 ± 0.026[a]
**Others (1)**								
VOC72	(9Z)-9-Tricosene	27519-02-4	$C_{23}H_{46}$	2277/2278	0.594 ± 0.233[c]	0.927 ± 0.077[b]	0.924 ± 0.080[b]	1.31 ± 0.19[a]
**Class**	**Acids**				1.16 ± 0.30[b]	0.956 ± 0.195[b]	1.26 ± 0.03[b]	2.63 ± 0.08[a]
	**Alcohols**				2.91 ± 0.43[d]	7.82 ± 1.05[a]	4.99 ± 0.19[c]	6.56 ± 0.31[b]
	**Aldehydes**				38.00 ± 7.76[b]	14.82 ± 2.36[c]	48.18 ± 5.73[a]	33.52 ± 1.72[b]
	**Esters**				7.27 ± 2.42[a]	5.95 ± 3.98[a]	5.03 ± 2.43[a]	3.20 ± 0.37[a]
	**Ethers**				0.225 ± 0.072[b]	0.202 ± 0.043[b]	0.306 ± 0.056[b]	1.08 ± 0.07[a]
	**Heterocyclics**				3.71 ± 0.53[b]	1.09 ± 0.17[c]	3.49 ± 0.28[b]	4.50 ± 0.29[a]
	**Ketones**				0.132 ± 0.028[c]	0.274 ± 0.056[b]	0.245 ± 0.011[b]	0.618 ± 0.026[a]
	**Terpenes**				10.82 ± 1.96[d]	16.61 ± 2.36[c]	21.91 ± 2.58[b]	43.75 ± 2.94[a]
	**Others**				0.594 ± 0.233[c]	0.927 ± 0.077[b]	0.924 ± 0.080[b]	1.31 ± 0.19[a]

Note:1. RI, retention index, calculated from the retention times of the compounds and a homologous series of n-alkanes (C7–C40) analyzed on an HP-5MS capillary column; RI ref., retention index obtained from the NIST database.

2.Relative contents are expressed as mean ± standard deviation (SD) (μg/g, n = 3). Different superscript letters within the same row indicate significant differences among drying methods according to Duncan's multiple range test ($p < 0.05$).

3."-" indicates that the compound was not detected under the corresponding drying condition. FD, freeze-drying; ID, indoor shade drying; SD, sun drying; HAD, hot-air drying.

Table S3. Identification of non-volatile metabolites in *Forsythia suspensa* flowers by LC–MS.

Compound ID	RT (min)	Compounds name	Class	Molecular Formula	Adduct Ion	Observed *m/z*	Error (ppm)	MS/MS	MSI Level
comp1	0.86	Lysine	Amino acid	$C_6H_{14}N_2O_2$	$[M+H]^+$	147.1128	0.15	130.086, 84.081, 85.084	Level 2
comp2	0.88	Histidine	Amino acid	$C_6H_9N_3O_2$	$[M+H]^+$	156.0767	-0.11	110.071, 95.06, 93.045	Level 2
comp3	0.89	Arginine	Amino acid	$C_6H_{14}N_4O_2$	$[M+H]^+$	175.1189	-0.13	158.093, 130.097, 116.071	Level 2
comp4	0.92	Glutamine	Amino acid	$C_5H_{10}N_2O_3$	$[M+H]^+$	147.0765	0.31	130.0497, 102.0548, 101.0708	Level 2
comp5	0.92	Asparagine	Amino acid	$C_4H_8N_2O_3$	$[M+H]^+$	133.0608	0.12	116.034, 88.039, 87.055	Level 2
comp6	0.93	Choline	Others	$C_5H_{13}NO$	$[M+H]^+$	104.1069	-0.82	87.044, 86.06, 60.081	Level 2
comp7	0.93	Pyroglutamic acid	Amino acid	$C_5H_7NO_3$	$[M+H]^+$	130.0499	0.26	84.0444, 83.0604, 56.0495	Level 2
comp8	0.94	Sorbitol	Sugars	$C_6H_{14}O_6$	$[M-H]^-$	181.0717	-0.22	163.0622, 101.0247, 89.0247	Level 2
comp9	0.94	Threonine	Amino acid	$C_4H_9NO_3$	$[M+H]^+$	120.0655	-0.12	102.0549, 74.06, 56.0495	Level 2
comp10	0.95	Glutamic acid	Amino acid	$C_5H_9NO_4$	$[M+H]^+$	148.0605	0.14	130.0499, 102.0549, 84.0444	Level 2
comp11	0.98	Threonic acid	Organic acid	$C_4H_8O_5$	$[M-H]^-$	135.0299	0.2	117.0197, 89.0246, 75.0089	Level 2
comp12	1.00	Quinic acid	Organic acid	$C_7H_{12}O_6$	$[M-H]^-$	191.0560	-0.49	173.0463, 127.0405	Level 2
comp13	1.00	Sucrose	Sugars	$C_{12}H_{22}O_{11}$	$[M-H]^-$	341.1083	-1.81	201.0716, 179.0568, 119.0352	Level 2
comp14	1.02	Malic acid	Organic acid	$C_4H_6O_5$	$[M-H]^-$	133.0143	0.14	115.0039, 89.0246, 71.014	Level 2
comp15	1.03	Adenosine	Others	$C_{10}H_{13}N_5O_4$	$[M+H]^+$	268.1041	0.24	136.0619, 119.0351, 94.0399	Level 2
comp16	1.34	Valine	Amino acid	$C_5H_{11}NO_2$	$[M+H]^+$	118.0862	-0.33	72.0807, 55.0542, 57.0572	Level 2
comp17	1.41	Citric acid	Organic acid	$C_6H_8O_7$	$[M-H]^-$	191.0197	-0.35	111.009, 87.0089, 85.0297	Level 2
comp18	1.52	cis-1-(β-D-Glucopyranosyloxy)-4-hydroxycyclohexaneacetic acid	CEDs	$C_{14}H_{24}O_9$	$[M-H]^-$	335.1347	-0.2	179.0575, 155.0719, 119.0353	Level 2
comp19	1.52	Rengyoside A	CEDs	$C_{14}H_{26}O_8$	$[M-H]^-$	321.1553	-0.45	321.1599, 203.0860, 201.0714	Level 2

comp20	1.62	cis-1,4-Dihydroxycyclohexaneacetic acid	CEDs	$C_8H_{14}O_4$	$[M-H]^-$	173.0819	-0.36	113.0607	Level 2
comp21	1.68	Tyrosine	Amino acid	$C_9H_{11}NO_3$	$[M+H]^+$	182.0812	-0.14	136.0756, 123.0439, 119.049	Level 2
comp22	1.70	Succinic acid	Organic acid	$C_4H_6O_4$	$[M-H]^-$	117.0194	0.32	99.0089, 73.0296, 55.0189	Level 2
comp23	1.78	2-(3,4-Dihydroxyphenyl) ethyl β-D-glucopyranoside	PhGs	$C_{14}H_{20}O_8$	$[M-H]^-$	315.1085	-0.27	153.0559, 135.0457, 89.0246	Level 2
comp24	1.80	Leucine	Amino acid	$C_6H_{13}NO_2$	$[M+H]^+$	132.1019	0.02	86.0964, 69.0699, 44.0495	Level 2
comp25	1.95	3-Hydroxy-3-methylglutaric acid	Organic acid	$C_6H_{10}O_5$	$[M-H]^-$	161.0456	0.03	99.0454, 101.0246	Level 2
comp26	2.44	4-Methoxybenzaldehyde	Others	$C_8H_8O_2$	$[M+H]^+$	137.0597	0.23	109.0647, 91.0542, 81.0699	Level 2
comp27	2.44	Cornoside	PhGs	$C_{14}H_{20}O_8$	$[M-H]^-$	315.1084	-0.38	203.0865, 201.0713, 135.0455, 119.0346, 101.0247	Level 1
comp28	2.94	Tyrosol	PhGs	$C_8H_{10}O_2$	$[M+H]^+$	139.0754	0.38	121.065, 111.0806, 97.0648	Level 2
comp29	2.98	Rengyoside B	CEDs	$C_{14}H_{24}O_8$	$[M-H]^-$	319.1397	-0.55	203.0862, 201.0712, 101.0246, 89.0246, 71.0140	Level 2
comp30	3.32	Phenylalanine	Amino acid	$C_9H_{11}NO_2$	$[M+H]^+$	166.0862	-0.33	120.0807, 103.0542, 91.0542	Level 2
comp31	3.37	Rengynic acid-1-O-β-D-glucopyranoside	CEDs	$C_{14}H_{24}O_9$	$[M-H]^-$	335.1347	-0.25	179.0579, 161.0463, 131.0347	Level 2
comp32	3.92	Forsythoside D	PhGs	$C_{20}H_{30}O_{13}$	$[M-H]^-$	477.1610	-0.87	315.1103, 179.0560, 161.0463, 135.0455	Level 2
comp33	4.03	1,5-Anhydro-6-O-(3,4,5-trihydroxybenzoyl)-D-glucitol	Organic acid	$C_{13}H_{16}O_9$	$[M-H]^-$	315.0721	-0.18	153.0191, 109.0298	Level 2
comp34	4.83	Vanillic acid	Organic acid	$C_8H_8O_4$	$[M-H]^-$	167.0350	0.09	123.0457, 108.022	Level 2
comp35	5.06	Hexose	Sugars	$C_6H_{12}O_6$	$[M-H]^-$	179.0561	-0.29	89.0244, 71.014, 59.0139	Level 2

comp36	5.84	trans-3-Indoleacrylic acid	Others	$C_{11}H_9NO_2$	$[M+H]^+$	188.0706	-0.21	146.06, 143.0729, 118.065	Level 2
comp37	5.97	4-Hydroxybenzoic acid	Organic acid	$C_7H_6O_3$	$[M-H]^-$	137.0244	0.15	109.0305, 93.0348	Level 2
comp38	5.97	Adoxosidic acid	Iridoids	$C_{16}H_{24}O_{10}$	$[M-H]^-$	375.1291	-1.46	337.1743, 213.0771, 201.0710, 169.0868, 151.0766	Level 1
comp39	6.24	5'-S-Methyl-5'-thioadenosine	Others	$C_{11}H_{15}N_5O_3S$	$[M+H]^+$	298.0968	-0.05	136.0619, 119.0352, 94.0401	Level 2
comp40	6.28	Forsythide	Iridoids	$C_{16}H_{22}O_{11}$	$[M-H]^-$	389.1084	-1.29	345.1214, 183.0665, 165.056, 121.0662, 119.0355	Level 2
comp41	6.55	Salidroside	PhGs	$C_{14}H_{20}O_7$	$[M-H]^-$	299.1135	-0.46	119.035, 101.0248	Level 1
comp42	6.71	Rebouoside B	PhGs	$C_{19}H_{28}O_{12}$	$[M-H]^-$	447.1504	-0.92	315.1107, 191.0566, 149.0456, 135.045, 131.0353	Level 2
comp43	7.05	Hydroxybenzoic acid	Organic acid	$C_7H_6O_3$	$[M-H]^-$	137.0244	0.16	93.0345	Level 2
comp44	7.14	Forsythoside E	PhGs	$C_{20}H_{30}O_{12}$	$[M-H]^-$	461.1660	-1.02	315.1081, 205.072, 163.0613, 153.0561, 135.0454	Level 1
comp45	7.31	Cistanoside F	PhGs	$C_{21}H_{28}O_{13}$	$[M-H]^-$	487.1454	-0.63	179.0350, 161.0246, 135.0453	Level 2
comp46	7.49	Benzyl-β-D-cellobioside	Organic acid	$C_{19}H_{28}O_{11}$	$[M-H]^-$	431.1555	-0.99	203.0869, 149.0460, 119.0506, 99.0091	Level 2
comp47	7.52	Hydroxytyrosol apiosylglucoside	PhGs	$C_{19}H_{28}O_{12}$	$[M-H]^-$	447.1504	-0.83	203.0868, 146.9616, 149.0457	Level 2
comp48	7.57	Caffeic acid glucoside	Organic acid	$C_{15}H_{18}O_9$	$[M-H]^-$	341.0872	-1.70	296.5141, 203.0867,179.0351, 135.0454	Level 2
comp49	7.72	Darendoside A	PhGs	$C_{19}H_{28}O_{11}$	$[M-H]^-$	431.1554	-1.02	299.1148, 203.0861, 201.0712	Level 2
comp50	7.74	Gardoside	Iridoids	$C_{16}H_{22}O_{10}$	$[M-H]^-$	374.1208	-1.39	165.0558, 150.0322, 201.0712	Level 2
comp51	7.81	Forsythenside B	CEDs	$C_{22}H_{26}O_{11}$	$[M-H]^-$	465.1398	-0.91	161.0456, 153.0552, 149.0247, 135.0454, 121.0297	Level 1

comp52	7.85	Chlorogenic acid	Organic acid	$C_{16}H_{18}O_9$	$[M-H]^-$	353.0873	-1.45	191.0562, 179.0361,161.0248, 135.0452	Level 2
comp53	7.91	p-Coumaric acid glucoside	Organic acid	$C_{15}H_{18}O_8$	$[M-H]^-$	325.0928	-0.19	163.0400, 145.0295, 119.0503	Level 2
comp54	7.98	Forsyxoside C	PhGs	$C_{29}H_{34}O_{14}$	$[M-H]^-$	605.1853	-3.69	397.1287, 375.1433, 327.1250, 297.1129, 203.0863	Level 2
comp55	8.15	Caffeic acid	Organic acid	$C_9H_8O_4$	$[M-H]^-$	179.0350	-0.13	135.0454, 117.0350, 91.0558	Level 2
comp56	8.44	Acylated disaccharide isomer	Sugars	$C_{17}H_{30}O_{12}$	$[M-H]^-$	425.1660	-0.98	203.0865, 201.0714, 161.0462, 149.0456, 119.0350	Level 3
comp57	8.49	Geniposide isomer	Iridoids	$C_{17}H_{24}O_{10}$	$[M-H]^-$	387.1292	-1.27	207.0664, 203.0865, 201.0712, 192.0431, 177.0195	Level 2
comp58	8.71	Forsythialan B	Lignans	$C_{21}H_{24}O_7$	$[M+H]^+$	389.1574	0.14	335.1281, 151.0752, 151.0385	Level 2
comp59	8.88	1-O-feruloyl-β-D-glucose	Organic acid	$C_{16}H_{20}O_9$	$[M-H]^-$	193.0505	-0.67	193.0508, 178.0273, 149.0610	Level 2
comp60	9.03	Hydroxyphenethyl diglycoside isomer	PhGs	$C_{19}H_{28}O_{12}$	$[M-H]^-$	447.1504	-0.86	269.1039, 203.0862, 161.0459	Level 3
comp61	9.07	Hydroxycyclohexanone glycoside isomer	CEDs	$C_{14}H_{24}O_8$	$[M-H]^-$	319.1397	-0.38	203.0864, 201.0713, 101.0247, 89.0250, 59.0138	Level 3
comp62	9.19	Benzyl diglycoside isomer	Organic acid	$C_{18}H_{26}O_{10}$	$[M-H]^-$	401.1449	-1.17	221.0820, 203.0863, 201.0710	Level 3
comp63	9.39	p-Coumaroylquinic acid I	Organic acid	$C_{16}H_{18}O_8$	$[M-H]^-$	337.0928	-0.15	191.0564, 173.0458, 163.0400, 93.0348	Level 2
comp64	9.48	Sinapic acid glucoside isomer	Organic acid	$C_{17}H_{22}O_{10}$	$[M-H]^-$	385.1136	-1.21	223.0614, 208.0377, 164.0477, 149.0245, 121.0297	Level 2
comp65	9.66	Asperuloside-type iridoid glycoside	Iridoids	$C_{18}H_{22}O_{11}$	$[M-H]^-$	413.1085	-1.02	267.0726, 203.0862, 201.0713, 163.0406, 119.0504	Level 3
comp66	9.70	Suspenoidside E	Iridoids	$C_{26}H_{32}O_{13}$	$[M-H]^-$	551.1768	-0.45	345.1339, 327.1238, 193.0508, 179.0366, 149.0614	Level 2

comp67	9.87	Caffeoyl phenylethanoid glycoside isomer I	PhGs	$C_{28}H_{34}O_{16}$	$[M-H]^-$	625.1774	-0.01	607.1711, 455.1209, 445.1333, 221.0468, 179.0352	Level 3
comp68	9.97	Forsythenside A	CEDs	$C_{22}H_{26}O_{10}$	$[M-H]^-$	449.1450	-0.81	151.0406, 135.0458, 133.03	Level 2
comp69	10.00	Methyl ester iridoid glycoside isomer	Iridoids	$C_{17}H_{24}O_{11}$	$[M-H]^-$	403.1241	-1.11	223.0614, 165.0557, 147.0451, 139.0403, 101.0245	Level 3
comp70	10.15	Feruloylquinic acid	Organic acid		$[M-H]^-$	367.1029	-1.40	191.0563, 173.0449, 134.0381, 93.0347	Level 2
comp71	10.21	Forsythoside C	PhGs	$C_{29}H_{36}O_{16}$	$[M-H]^-$	639.1932	0.21	477.1617, 161.0246, 135.0454	Level 2
comp72	10.39	β-Hydroxyacteoside	PhGs	$C_{29}H_{36}O_{16}$	$[M-H]^-$	639.1932	0.21	621.1813, 459.1513, 179.0351, 161.0245, 151.0402	Level 2
comp73	10.40	Acetylated lignan glycoside	Lignans	$C_{32}H_{42}O_{16}$	$[M-H]^-$	681.2401	0.06	357.1341, 203.0865, 201.0715, 151.0402, 136.0169	Level 3
comp74	10.41	p-Coumaroylquinic acid II	Organic acid	$C_{16}H_{18}O_8$	$[M-H]^-$	337.0929	-0.04	191.0561, 173.0455, 163.0402, 119.0503, 93.0347	Level 2
comp75	10.47	2-Hydroxycyclohexyl caffeoyl hexoside	CEDs	$C_{21}H_{28}O_{10}$	$[M-H]^-$	439.1606	-0.89	203.0863, 201.0711, 137.0246, 93.0347, 59.0138	Level 3
comp76	10.47	β-Hydroxyacteoside isomer	PhGs	$C_{29}H_{36}O_{16}$	$[M-H]^-$	639.1932	0.23	621.1880, 161.0246, 151.0401, 179.0349, 135.0450	Level 2
comp77	10.52	Forsythoside M	PhGs	$C_{22}H_{26}O_{10}$	$[M-H]^-$	449.1450	-0.83	315.1117, 205.0508, 193.0507, 161.0609, 151.0402	Level 2
comp78	10.69	Phenethyl β-primeveroside	PhGs	$C_{19}H_{28}O_{10}$	$[M-H]^-$	415.1606	-0.99	131.0352, 113.0247, 101.0245, 89.0245, 71.0140	Level 2
comp79	10.75	Benzoylated iridoid glycoside	Iridoids	$C_{22}H_{28}O_{10}$	$[M-H]^-$	451.1605	-0.98	161.0462, 151.0403, 123.0449, 119.0355, 113.0253	Level 3
comp80	10.79	Kaempferol O-dihexoside-rhamnoside isomer I	Flavonoids	$C_{33}H_{40}O_{20}$	$[M-H]^-$	755.2040	-0.04	300.0279, 284.0320, 255.0303, 243.0305, 227.0355	Level 2

comp81	10.89	Rengyoside D	CEDs	$C_{22}H_{30}O_{11}$	$[M-H]^-$	469.1712	-0.79	193.0505, 161.0617, 151.0403	Level 2
comp82	10.97	Calceolarioside A	PhGs	$C_{23}H_{26}O_{11}$	$[M-H]^-$	477.1401	-0.4	179.0354, 161.025, 135.0456	Level 2
comp83	10.79	Kaempferol O-dihexoside-rhamnoside isomer II	Flavonoids	$C_{33}H_{40}O_{20}$	$[M-H]^-$	755.2040	-0.04	575.1423, 284.0330, 255.0300, 227.0351, 183.0452	Level 2
comp84	11.26	Forsythialanside E	Lignans	$C_{26}H_{32}O_{11}$	$[M-H]^-$	535.1819	-0.32	343.1188, 313.1085, 181.0507, 151.0402	Level 2
comp85	11.31	Caffeoyl phenylethanoid glycoside isomer II	PhGs	$C_{29}H_{34}O_{16}$	$[M-H]^-$	637.1773	-0.24	475.1466, 457.1373, 203.0868, 201.0707, 161.0246	Level 3
comp86	11.40	Secoisolariciresinol glucoside	Lignans	$C_{26}H_{36}O_{11}$	$[M-H]^-$	523.2183	-0.34	361.1664, 203.0871, 201.0714, 165.0560, 121.0297	Level 2
comp87	11.44	Lariciresinol 4-O-β-D-glucopyranoside	Lignans	$C_{26}H_{34}O_{11}$	$[M-H]^-$	521.2026	-0.44	329.14, 161.0608, 147.0455, 131.0507, 121.0296	Level 2
comp88	11.50	Plantamajoside	PhGs	$C_{29}H_{36}O_{16}$	$[M-H]^-$	639.1933	0.37	179.0359, 161.0245, 151.0401, 135.0452, 133.0298	Level 2
comp89	11.63	Forsythoside I	PhGs	$C_{29}H_{36}O_{15}$	$[M-H]^-$	623.1981	-0.02	461.1656, 443.1554, 179.035, 161.0245, 153.0559	Level 2
comp90	11.66	Forsythoside I isomer	Flavonoids	$C_{26}H_{32}O_{11}$	$[M-H]^-$	581.1877	0.22	475.1466, 203.0868, 201.0707, 161.0246	Level 2
comp91	11.75	Forsythoside J	PhGs	$C_{28}H_{34}O_{15}$	$[M-H]^-$	609.1823	-0.3	447.1542, 161.0246, 179.0354, 135.0451, 133.0298	Level 2
comp92	11.97	Rutin	Flavonoids	$C_{27}H_{30}O_{16}$	$[M-H]^-$	609.1467	0.96	301.0349, 300.0278, 271.0251, 255.0302, 151.0039	Level 1
comp93	12.00	Calceolarioside C	PhGs	$C_{28}H_{34}O_{15}$	$[M-H]^-$	609.1807	-2.99	447.1517, 300.0276, 271.0251, 255.0300, 161.0245	Level 2
comp94	12.20	Isoforsythiaside A	PhGs	$C_{29}H_{36}O_{15}$	$[M-H]^-$	623.1982	0.16	461.1692, 443.1567, 179.0353, 161.0246, 135.0454	Level 1

comp95	12.36	Quercetin-O-disaccharide isomer	Flavonoids	$C_{27}H_{30}O_{16}$	$[M-H]^-$	609.1460	-0.25	300.0278, 301.0349, 271.0252, 255.0302, 243.0300	Level 2
comp96	12.71	S-Suspensaside methyl ether	Lignans	$C_{30}H_{38}O_{16}$	$[M-H]^-$	653.2089	0.26	621.1821, 161.0247, 179.0352, 135.0455, 133.0298	Level 2
comp97	12.71	Forsyoxaside F	PhGs	$C_{32}H_{36}O_{16}$	$[M-H]^-$	675.1906	-3.56	643.1635, 203.0864, 201.0711, 179.0354, 161.0243	Level 2
comp98	12.79	Fraxiresinol-4′-O-β-D-glucopyranoside	Lignans	$C_{27}H_{34}O_{13}$	$[M-H]^-$	565.1927	-0.02	357.1336, 342.11, 327.1258, 163.0412, 151.0403	Level 2
comp99	12.82	Plantainoside A	PhGs	$C_{23}H_{26}O_{11}$	$[M-H]^-$	477.1399	-0.78	315.1065, 161.0245, 201.0710, 179.0353, 135.0454	Level 2
comp100	12.93	Hyperoside	Flavonoids	$C_{21}H_{20}O_{12}$	$[M-H]^-$	463.0878	-0.83	301.0348, 300.0276, 271.0248, 255.0297, 151.0036	Level 2
comp101	13.48	Adoxosidic acid-6′-oleuroperic ester	Iridoids	$C_{30}H_{36}O_{16}$	$[M-H]^-$	651.1929	-0.18	221.0459, 179.0347, 161.0246, 135.0453, 133.0296	Level 2
comp102	13.52	Suspensaside A	Lignans	$C_{29}H_{34}O_{15}$	$[M-H]^-$	621.1836	1.79	477.1406, 161.0246, 179.0358, 201.0714, 135.0456	Level 2
comp103	13.52	Forsythoside A	PhGs	$C_{29}H_{36}O_{15}$	$[M-H]^-$	623.1981	-0.06	461.1676, 443.1559, 201.0717, 179.0352, 161.0247	Level 1
comp104	13.68	Kaempferol 3-O-rutinoside	Flavonoids	$C_{27}H_{30}O_{15}$	$[M-H]^-$	593.1513	0.09	285.0403, 284.0327, 255.0300, 227.0350, 201.0713	Level 2
comp105	13.97	Lianqiaoxinoside C	PhGs	$C_{28}H_{34}O_{15}$	$[M-H]^-$	609.1824	-0.17	447.1505, 271.0265, 179.0361, 161.0246, 135.0454	Level 2
comp106	14.21	Calceolarioside B	PhGs	$C_{23}H_{26}O_{11}$	$[M-H]^-$	477.1400	-0.58	315.1085, 281.0664, 221.0439, 179.0352, 161.0246	Level 1
comp107	14.66	Forsythenside J	CEDs	$C_{24}H_{28}O_{11}$	$[M-H]^-$	491.1556	-0.59	345.4548, 235.0624, 193.0516, 175.04, 161.0225	Level 2

comp108	14.84	Apiosyl-caffeoyl phenylethanoid glycoside	PhGs	$C_{28}H_{34}O_{14}$	$[M-H]^-$	593.1877	0.2	447.1539, 429.1393, 285.0403, 163.0404, 145.0302	Level 3
comp109	15.00	Swertiamacroside	PhGs	$C_{21}H_{28}O_{13}$	$[M-H]^-$	487.1357	-0.31	163.0403, 145.0297, 119.0504	Level 2
comp110	15.31	(+)-Pinoresinol 4′-O-β-D-glucopyranoside	Lignans	$C_{26}H_{32}O_{11}$	$[M-H]^-$	519.1869	-0.52	357.1344, 342.1115, 311.1326, 151.0402, 136.0167	Level 1
comp111	15.45	Kaempferol O-disaccharide isomer	Flavonoids	$C_{27}H_{30}O_{15}$	$[M-H]^-$	593.1511	-0.12	285.0403, 284.0328, 255.0300, 227.0351, 201.0717	Level 2
comp112	16.29	Isoacteoside	PhGs	$C_{29}H_{36}O_{15}$	$[M-H]^-$	623.1981	-0.02	461.1666, 443.1525, 203.0862, 179.0351, 161.0245	Level 1
comp113	16.55	Narcissoside	Flavonoids	$C_{28}H_{32}O_{16}$	$[M-H]^-$	623.1622	0.68	315.0510, 299.0201, 271.0250, 255.0299	Level 2
comp114	16.72	Campneoside I	PhGs	$C_{30}H_{38}O_{16}$	$[M-H]^-$	653.2088	0.2	491.1758, 476.1519, 179.0352, 161.0245, 135.0453	Level 2
comp115	17.66	Koreanaside A	Lignans	$C_{26}H_{32}O_{11}$	$[M-H]^-$	519.1871	-0.09	357.1334, 342.11, 163.0412	Level 2
comp116	17.87	Forsythenside K	PhGs	$C_{29}H_{36}O_{14}$	$[M-H]^-$	607.2034	0.26	461.1688, 443.1564, 163.0399,135.0452	Level 2
comp117	18.13	8-Hydroxypinoresinol	Lignans	$C_{20}H_{22}O_7$	$[M-H]^-$	373.1288	-1.28	358.1058, 343.1223, 313.1086, 163.0401	Level 2
comp118	19.19	Aloin B	Flavonoids	$C_{21}H_{22}O_9$	$[M-H]^-$	417.1187	-1.1	255.066, 153.0202, 135.0088, 119.0504, 103.6499	Level 2
comp119	22.60	Phillygenin	Lignans	$C_{21}H_{24}O_6$	$[M-H]^-$	579.2083	-0.06	371.1498, 356.1274, 201.0723, 121.0296	Level 1
comp120	22.72	Suspenoidside C	Iridoids	$C_{25}H_{30}O_{12}$	$[M-H]^-$	521.1663	-0.34	315.1262, 203.0865, 201.0710, 163.0404	Level 2
comp121	23.36	Simplocosin	Lignans	$C_{26}H_{32}O_{11}$	$[M-H]^-$	519.1870	-0.35	357.1311, 203.0862, 201.0710, 151.0401, 136.0168	Level 2

comp122	23.59	Suspenoidside B	Iridoids	$C_{25}H_{30}O_{12}$	$[M-H]^-$	521.1663	-0.37	315.1243, 297.1137, 203.0868, 163.0404	Level 2
comp123	23.72	Acanthoside B	Lignans	$C_{28}H_{36}O_{13}$	$[M-H]^-$	579.2083	0.04	371.1516, 356.1279, 203.0864, 201.0711	Level 2
comp124	24.15	Pinoresinol	Lignans	$C_{20}H_{22}O_6$	$[M+H]^+$	359.1490	0.2	323.1274, 291.1014, 271.0965, 248.0834, 203.0852	Level 1
comp125	25.36	Arctigenin	Lignans	$C_{21}H_{24}O_6$	$[M-H]^-$	371.1494	-1.71	151.0764, 136.0529, 121.0295	Level 1
comp126	26.35	Forsythenside L	CEDs	$C_{20}H_{28}O_{11}$	$[M-H]^-$	443.1556	-0.66	203.0863, 201.0702, 102.9566	Level 2
comp127	26.79	Forsypensin C	Terpenes	$C_{20}H_{30}O_5$	$[M+H]^+$	351.2143	-0.01	333.2062, 319.4277, 161.0962, 145.1017, 121.065	Level 2
comp128	26.87	Suspenoidsides D	Iridoids	$C_{25}H_{30}O_{13}$	$[M-H]^-$	537.1566	-1.42	243.0665, 137.025, 135.0089, 109.0298, 108.0219	Level 2
comp129	28.31	Forsypensin E	Terpenes	$C_{20}H_{28}O_5$	$[M+H]^+$	349.1986	-0.17	285.1856, 267.1732, 191.1433, 163.0745, 135.0804	Level 2
comp130	31.28	Wilforlide B	Terpenes	$C_{30}H_{46}O_4$	$[M+H]^+$	453.3365	0.34	407.3322, 219.1744, 201.1641	Level 2
comp131	32.27	Esculentic acid	Terpenes	$C_{30}H_{48}O_5$	$[M+H]^+$	489.3577	-0.17	435.3245, 407.3309, 389.3226, 219.1744, 201.1638	Level 2
comp132	32.90	Phytosphingosine	Lipids	$C_{18}H_{39}NO_3$	$[M+H]^+$	318.3003	0.12	300.2897, 282.2794, 270.2795	Level 2
comp133	33.02	Eicosapentaenoic acid	Organic acid	$C_{20}H_{30}O_2$	$[M+H]^+$	303.2319	-0.1	285.2207, 257.226, 243.211	Level 2
comp134	35.62	Oleanonic acid	Terpenes	$C_{30}H_{46}O_3$	$[M+H]^+$	455.3522	0.42	409.3506, 203.179, 189.1639, 119.0856, 95.0856	Level 2
comp135	35.76	18-β-Glycyrrhetinic acid	Terpenes	$C_{30}H_{46}O_4$	$[M+H]^+$	471.3470	-0.33	425.3417, 407.3317, 317.2097, 271.2069, 235.169	Level 2
comp136	35.88	α-Linolenic acid	Organic acid	$C_{18}H_{30}O_2$	$[M+H]^+$	279.2319	0.14	191.1429, 173.1323, 149.0233	Level 2
comp137	36.78	Corosolic acid	Terpenes	$C_{30}H_{48}O_4$	$[M+H]^+$	473.3627	-0.14	205.1587, 189.1662, 107.0855	Level 2

comp138	37.07	18-Hydroxyretinoic acid	Terpenes	$C_{20}H_{28}O_3$	$[M+H]^+$	317.2088	-0.17	299.2001, 289.2176, 271.2058, 189.1639, 161.1327	Level 2
comp139	38.56	Linolenelaidic acid	Organic acid	$C_{18}H_{30}O_2$	$[M+H]^+$	279.2318	0.23	149.0232, 137.1322, 109.101	Level 2
comp140	38.71	Maslinic acid	Terpenes	$C_{30}H_{48}O_4$	$[M+H]^+$	473.3627	0.1	437.3422, 427.3572, 409.3457, 391.3369, 357.2803	Level 2
comp141	41.65	betulonic acid	Terpenes	$C_{30}H_{46}O_3$	$[M+H]^+$	455.3522	-0.1	391.3344, 285.1811, 215.1797, 189.1639, 175.1474	Level 2
comp142	41.83	Rubicoumaric acid	Terpenes	$C_{39}H_{54}O_6$	$[M+H]^+$	619.3997	0.05	437.3409, 409.3487, 391.336, 215.1788, 191.18	Level 2
comp143	42.90	Oleana-2,12-dien-28-oic acid	Terpenes	$C_{30}H_{46}O_2$	$[M+H]^+$	439.3572	-0.44	393.3519, 269.2265, 203.1798, 179.1798, 175.1484	Level 2
comp144	43.50	Betulinic acid	Terpenes	$C_{30}H_{48}O_3$	$[M+H]^+$	457.3677	-0.26	393.3519, 249.1854, 205.1949, 191.1792, 189.1635	Level 2
comp145	43.51	Olean-12-ene-3,11-dione	Terpenes	$C_{30}H_{46}O_2$	$[M+H]^+$	439.3571	-0.26	393.3537, 249.186, 215.18, 205.1956, 191.1791	Level 2
comp146	44.04	β-Amyrone	Terpenes	$C_{30}H_{48}O$	$[M+H]^+$	425.3780	-0.04	407.3679, 163.1478, 257.227, 191.1801, 161.133	Level 2
comp147	45.70	Oleamide	Lipids	$C_{18}H_{35}NO$	$[M+H]^+$	282.2791	-0.11	265.2527, 247.242, 128.1066	Level 2
comp148	48.83	Lupeol	Terpenes	$C_{30}H_{50}O$	$[M+H]^+$	427.3935	-0.24	409.3828, 219.2109, 205.1955, 191.1797, 161.1326	Level 2

Note: PhGs, phenylethanoid glycosides; CEDs, cyclohexyl ethanol derivatives.

Table S4. Table S4. Differential volatile and non-volatile compounds screened based on VIP > 1 and q-value < 0.05 across the four drying treatments.

Platform	No.	Compound	Class	VIP value	q-value
HS-GC-MS	1	Pyranone	Heterocyclics	1.82	1.14E-05
	2	(Z)-linalool oxide (pyranoid)	Heterocyclics	1.73	3.96E-05
	3	Myristic acid	Acids	1.61	8.13E-03
	4	(R)-(-)-1,2-Propanediol	Alcohols	1.49	1.38E-05
	5	Benzyl alcohol	Alcohols	1.43	2.08E-03
	6	Camphene	Terpenes	1.35	1.68E-03
	7	3-Hexen-1-ol	Alcohols	1.32	6.56E-05
	8	Methyl decanoate	Esters	1.29	8.58E-03
	9	Furfural	Heterocyclics	1.22	1.81E-05
	10	Ethyl octadecanoate	Esters	1.21	2.50E-06
	11	Hexadecanal	Aldehydes	1.18	1.00E-04
	12	pentadecanal	Aldehydes	1.16	1.92E-04
	13	Dodecanal	Aldehydes	1.13	6.56E-05
	14	Terpinen-4-ol	Terpenes	1.08	9.60E-03
	15	α-cadinol	Alcohols	1.06	1.70E-04
	16	1-Hexanol	Alcohols	1.06	3.63E-03
	17	β-Pinene	Terpenes	1.03	1.48E-05
**LC-MS**	1	2-(3,4-Dihydroxyphenyl) ethyl β-D-glucopyranoside	PhGs	1.69	2.46E-06
	2	Adoxosidic acid-6′-oleuroperic ester	Iridoids	1.68	8.83E-05
	3	Cornoside	PhGs	1.65	6.29E-06
	4	Eicosapentaenoic acid	Organic acid	1.63	1.33E-04

5	Tyrosol	PhGs	1.61	9.54E-08
6	Rengyoside B	CEDs	1.6	6.16E-06
7	Forsythenside L	CEDs	1.57	2.61E-06
8	Rubicoumaric acid	Terpenes	1.54	4.17E-03
9	betulonic acid	Terpenes	1.5	1.74E-03
10	Histidine	Amino acid	1.49	2.61E-06
11	Swertiamacroside	PhGs	1.47	4.32E-06
12	Olean-12-ene-3,11-dione	Terpenes	1.47	1.94E-03
13	Esculentic acid	Terpenes	1.46	5.84E-08
14	Succinic acid	Organic acid	1.45	6.16E-06
15	Pinoresinol	Lignans	1.44	1.06E-03
16	Valine	Amino acid	1.41	2.72E-08
17	Forsythoside D	PhGs	1.39	1.14E-04
18	Arginine	Amino acid	1.38	5.37E-05
19	Plantainoside A	PhGs	1.37	1.97E-05
20	cis-1,4-Dihydroxycyclohexaneacetic acid	CEDs	1.36	3.83E-05
21	Oleanonic acid	Terpenes	1.36	5.79E-04
22	Forsythoside I isomer	Flavonoids	1.35	3.28E-05
23	Betulinic acid	Terpenes	1.34	2.15E-03
24	Suspenoidside E	Iridoids	1.32	4.76E-05
25	Sorbitol	Sugars	1.3	7.09E-10
26	18-β-Glycyrrhetinic acid	Terpenes	1.29	4.32E-04
27	Hydroxytyrosol apiosylglucoside	PhGs	1.26	6.92E-06
28	18-Hydroxyretinoic acid	Terpenes	1.25	5.38E-06

29	Forsythialanside E	Lignans	1.22	5.37E-05
30	Forsypensin E	Terpenes	1.21	1.79E-04
31	Forsythenside B	CEDs	1.2	1.28E-05
32	Suspenoidsides D	Iridoids	1.2	5.33E-04
33	5′-S-Methyl-5′-thioadenosine	Others	1.2	3.94E-06
34	Salidroside	PhGs	1.19	1.87E-05
35	Plantamajoside	PhGs	1.19	1.03E-04
36	Citric acid	Organic acid	1.17	6.11E-05
37	Geniposide isomer	Iridoids	1.17	6.91E-06
38	Simplocosin	Lignans	1.16	1.15E-04
39	Phillygenin	Lignans	1.14	3.97E-04
40	Corosolic acid	Terpenes	1.13	5.37E-05
41	Forsythenside A	CEDs	1.12	3.57E-03
42	Benzoylated iridoid glycoside isomer	Iridoids	1.12	8.15E-05
43	Forsythoside M	PhGs	1.12	5.37E-05
44	Gardoside	Iridoids	1.11	3.10E-07
45	1,5-Anhydro-6-O-(3,4,5-trihydroxybenzoyl)-D-glucitol	Organic acid	1.11	3.69E-04
46	Campneoside I	PhGs	1.11	1.42E-04
47	Pyroglutamic acid	Amino acid	1.11	1.28E-05
48	Malic acid	Organic acid	1.1	1.32E-04
49	p-Coumaroylquinic acid I	Organic acid	1.1	8.24E-04
50	2-Hydroxycyclohexyl caffeoyl hexoside	CEDs	1.1	6.03E-04
51	Forsypensin C	Terpenes	1.08	2.17E-06
52	Acanthoside B	Lignans	1.08	1.33E-04

53	Asperuloside-type iridoid glycoside	Iridoids	1.08	6.05E-04
54	Hydroxybenzoic acid	Organic acid	1.05	9.65E-05
55	Kaempferol O-dihexoside-rhamnoside isomer I	Flavonoids	1.05	4.34E-04
56	Kaempferol O-dihexoside-rhamnoside isomer II	Flavonoids	1.05	4.34E-04
57	Aloin B	Flavonoids	1.05	1.90E-06
58	Tyrosine	Amino acid	1.04	7.21E-05
59	Calceolarioside B	PhGs	1.04	1.04E-02
60	trans-3-Indoleacrylic acid	Others	1.03	1.17E-03
61	Forsyoxaside F	PhGs	1.01	8.13E-03
62	S-Suspensaside methyl ether	Lignans	1	5.97E-03

Note: Compounds are grouped by analytical platform and sorted by VIP value in descending order within each platform. q-values represent FDR-adjusted p-values.

Table S5. Sensory evaluation scores of *Forsythia suspensa* flower infusions under different drying treatments.

Attribute	FD	ID	SD	HAD
Appearance	7.02 ± 0.15^{a}	6.22 ± 0.35^{b}	4.88 ± 0.23^{d}	5.48 ± 0.52^{c}
Infusion color	3.77 ± 0.29^{c}	4.60 ± 0.38^{b}	6.80 ± 0.37^{a}	6.45 ± 0.35^{a}
Aroma	5.17 ± 0.33^{b}	5.63 ± 0.40^{b}	6.73 ± 0.63^{a}	6.42 ± 0.19^{a}
Taste	3.58 ± 0.29^{d}	4.45 ± 0.41^{c}	5.23 ± 0.36^{b}	6.78 ± 0.37^{a}
Overall acceptability	6.42 ± 0.35^{a}	6.13 ± 0.24^{a}	5.73 ± 0.45^{b}	5.97 ± 0.27^{a}

Note: Each value is expressed as the mean ± SD (n = 6). Different superscript letters within the same row indicate significant differences between drying treatments according to Duncan's multiple range test ($p < 0.05$).
